# Healthy lifestyle and life expectancy at age 30 in the Chinese population: an observational study

**DOI:** 10.1016/S2468-2667(22)00110-4

**Published:** 2022-08-01

**Authors:** Qiufen Sun, Dongmei Yu, Junning Fan, Canqing Yu, Yu Guo, Pei Pei, Ling Yang, Yiping Chen, Huaidong Du, Xiaoming Yang, Sam Sansome, Yongming Wang, Wenhua Zhao, Junshi Chen, Zhengming Chen, Liyun Zhao, Jun Lv, Liming Li, Members of the China Kadoorie Biobank collaborative group, International Steering Committee, Junshi Chen, Zhengming Chen, Rory Collins, Liming Li, Richard Peto, International Co-ordinating Centre Oxford, Daniel Avery, Ruth Boxall, Derrick Bennett, Yumei Chang, Yiping Chen, Zhengming Chen, Robert Clarke, Huaidong Du, Simon Gilbert, Alex Hacker, Mike Hill, Michael Holmes, Andri Iona, Christiana Kartsonaki, Rene Kerosi, Ling Kong, Om Kurmi, Garry Lancaster, Sarah Lewington, Kuang Lin, John McDonnell, Iona Millwood, Qunhua Nie, Jayakrishnan Radhakrishnan, Sajjad Rafiq, Paul Ryder, Sam Sansome, Dan Schmidt, Paul Sherliker, Rajani Sohoni, Becky Stevens, Iain Turnbull, Robin Walters, Jenny Wang, Lin Wang, Neil Wright, Ling Yang, Xiaoming Yang, National Co-ordinating Centre, Beijing, Zheng Bian, Yu Guo, Xiao Han, Can Hou, Jun Lv, Pei Pei, Yunlong Tan, Canqing Yu, 10 Regional Co-ordinating Centres: Qingdao CDC, Zengchang Pang, Ruqin Gao, Shanpeng Li, Shaojie Wang, Yongmei Liu, Ranran Du, Yajing Zang, Liang Cheng, Xiaocao Tian, Hua Zhang, Yaoming Zhai, Feng Ning, Xiaohui Sun, Feifei Li, Licang CDC, Silu Lv, Junzheng Wang, Wei Hou, Heilongjiang Provincial CDC, Mingyuan Zeng, Ge Jiang, Xue Zhou, Nangang CDC, Liqiu Yang, Hui He, Bo Yu, Yanjie Li, Qinai Xu, Quan Kang, Ziyan Guo, Hainan Provincial CDC, Dan Wang, Ximin Hu, Hongmei Wang, Jinyan Chen, Yan Fu, Zhenwang Fu, Xiaohuan Wang, Meilan CDC, Min Weng, Zhendong Guo, Shukuan Wu, Yilei Li, Huimei Li, Zhifang Fu, Jiangsu Provincial CDC, Ming Wu, Yonglin Zhou, Jinyi Zhou, Ran Tao, Jie Yang, Jian Su, Suzhou CDC, Fang liu, Jun Zhang, Yihe Hu, Yan Lu, Liangcai Ma, Aiyu Tang, Shuo Zhang, Jianrong Jin, Jingchao Liu, Guangxi Provincial CDC, Zhenzhu Tang, Naying Chen, Ying Huang, Liuzhou CDC, Mingqiang Li, Jinhuai Meng, Rong Pan, Qilian Jiang, Jian Lan, Yun Liu, Liuping Wei, Liyuan Zhou, Ningyu Chen Ping Wang, Fanwen Meng, Yulu Qin, Sisi Wang, Sichuan Provincial CDC, Xianping Wu, Ningmei Zhang, Xiaofang Chen, Weiwei Zhou, Pengzhou CDC, Guojin Luo, Jianguo Li, Xiaofang Chen, Xunfu Zhong, Jiaqiu Liu, Qiang Sun, Gansu Provincial CDC, Pengfei Ge, Xiaolan Ren, Caixia Dong, Maiji CDC, Hui Zhang, Enke Mao, Xiaoping Wang, Tao Wang, Xi zhang, Henan Provincial CDC, Ding Zhang, Gang Zhou, Shixian Feng, Liang Chang, Lei Fan, Huixian CDC, Yulian Gao, Tianyou He, Huarong Sun, Pan He, Chen Hu, Xukui Zhang, Huifang Wu, Pan He, Zhejiang Provincial CDC, Min Yu, Ruying Hu, Hao Wang, Tongxiang CDC, Yijian Qian, Chunmei Wang, Kaixu Xie, Lingli Chen, Yidan Zhang, Dongxia Pan, Qijun Gu, Hunan Provincial CDC, Yuelong Huang, Biyun Chen, Li Yin, Huilin Liu, Zhongxi Fu, Qiaohua Xu, Liuyang CDC, Xin Xu, Hao Zhang, Huajun Long, Xianzhi Li, Libo Zhang, Zhe Qiu

**Affiliations:** 1 Department of Epidemiology & Biostatistics, School of Public Health, Peking University, Beijing 100191, China; 2 National Institute for Nutrition and Health, Chinese Center for Disease Control and Prevention, Beijing, China; 3 Peking University Center for Public Health and Epidemic Preparedness & Response, Beijing 100191, China; 4 Fuwai Hospital Chinese Academy of Medical Sciences, Beijing, China;; 5 Chinese Academy of Medical Sciences, Beijing, China; 6 Medical Research Council Population Health Research Unit at the University of Oxford, Oxford, United Kingdom; 7 Clinical Trial Service Unit & Epidemiological Studies Unit (CTSU), Nuffield Department of Population Health, University of Oxford, United Kingdom; 8 NCDs Prevention and Control Department, Maiji CDC, Tianshui, Gansu, China; 9 China National Center for Food Safety Risk Assessment, Beijing, China; 10 Key Laboratory of Molecular Cardiovascular Sciences (Peking University), Ministry of Education, Beijing, China

## Abstract

**Background:**

The improvement of life expectancy is one of the aims of the “Healthy China 2030” blueprint. We aimed to investigate the extent to which healthy lifestyles are associated with life expectancy in Chinese adults.

**Methods:**

We used the prospective study of China Kadoorie Biobank (CKB) study (n=487,209) to examine the relative risk of mortality associated with individual and combined lifestyle factors (never smoking or quitting not for illness, no excessive alcohol use, being physically active, healthy eating habits, and healthy body shape). We estimated the national prevalence of lifestyle factors using data from the China Nutrition and Health Surveillance (2015) and derived mortality rates from the Global Burden of Diseases, Injuries, and Risk Factors Study (2015). All three data sources were combined to estimate the life expectancy of individuals at age 30 following different levels of lifestyle factors by using life table method. The cause-specific decomposition of the life expectancy differences was analyzed using Arriaga’s method.

**Findings:**

There were 42,496 deaths documented over a median follow-up of 11.1 (interquartile range: 10.2-12.1) years in CKB. The adjusted hazard ratios (95% confidence intervals [CIs]) of participants adopting five versus 0-1 low-risk factors was 0.38 (0.34, 0.43) for all-cause mortality, 0.37 (0.30, 0.46) for cardiovascular disease (CVD) mortality, 0.47 (0.39, 0.56) for cancer mortality, and 0.30 (0.14, 0.64) for chronic respiratory disease (CRD) mortality. The life expectancy (95%CI) at age 30 for individuals with 0-1 low-risk factor was on average 41.7 (41.5, 42.0) years for men and 47.3 (46.6, 48.0) years for women. When individuals adopted all five low-risk factors, the life expectancy was 50.5 (48.5, 52.4) years for men and 55.4 (53.5, 57.4) years for women, with an increase (95%CI) of 8.8 (6.8, 10.7) years (men) and 8.1 (6.5, 9.9) years (women), respectively. The estimated extended life expectancy for men and women was attributable to reduced death from CVD (2.4 years [27% out of the total extended life expectancy] for men and 3.6 years [46%] for women), cancer (2.5 years [29%] and 0.9 years [11%]), and CRD (0.6 years [7%] and 1.3 years [16%]).

**Interpretation:**

Our findings suggest that increasing the adoption of these five healthy lifestyle factors through public health interventions could be associated with substantial gains in life expectancy in the Chinese population.

## Introduction

Traditional lifestyle-related risk factors, including smoking, overdrinking, physical inactivity, poor dietary habits, and obesity, have been associated with increased risk of death, especially from chronic diseases.^1,2^ The widespread prevalence of these risk factors has caused a great burden of disease worldwide,^3^ with no exception to China.^4^ Professional indicators, like the relative risk and the absolute lifetime risk, might be a little hard to understand for laypeople. In contrast, life expectancy being an absolute quantitative measure is more intuitive and has become a common metric for establishing public health priorities.

The available studies that assessed the relationship between lifestyle and life expectancy were mainly performed in the North American and European populations, suggesting that healthier lifestyles were associated with an increase in life expectancy of 7.4 to 18.5 years.^5–7^ Most of these studies were based on specific cohort populations; the results of such a study design only reflect the mortality level of specific cohort populations over a follow-up period and should be cautious in generalizing to the national population.

There are non-negligible differences between Chinese and Western populations in economic and social development and determinants of health, such as genetics, lifestyle, and hazardous environmental exposures. However, only few studies have evaluated the impact of individual lifestyle factors, such as smoking and alcohol intake, on the life expectancy of the Chinese population.^8–10^ The impact of combined lifestyle behaviors on Chinese life expectancy remains unclear, and the evidence gaps need to be filled.

The blueprint of Healthy China 2030 set out the goal of increasing the average life expectancy of Chinese people at birth from 76.3 years in 2015 to 79 years in 2030. We, therefore, aimed to evaluate the potential impacts of individual and combined low-risk lifestyle factors on the life expectancy at 30 years in the Chinese population.

## Methods

We combined three sources of data (Figure S1): (1) the China Kadoorie Biobank (CKB) study for the association between lifestyle factors and mortality; (2) the Global Burden of Diseases, Injuries, and Risk Factors Study (GBD, 2015) for population-based mortality rates; and (3) the China Nutrition and Health Surveillance (CNHS, 2015) for population-based prevalence of lifestyle factors.

### Study design and participants

The CKB study is a nationwide population-based prospective cohort of over 0.5 million adults. Details of the study design have been described elsewhere.^11^ Briefly, 512,725 participants aged 30-79 years were recruited during 2004-2008 from five urban and five rural areas geographically spread across China. Baseline survey and anthropometric measurements were undertaken by trained study staff. All participants signed informed consent forms. Ethical approval was obtained from the Ethics Review Committee of the Chinese Center for Disease Control and Prevention (CDC; Beijing, China) and the Oxford Tropical Research Ethics Committee, University of Oxford (UK).

In the present study, participants with coronary heart disease (n=15,472), stroke (n=8,884), or cancer (n=2,578) at baseline were excluded, as well as two people with missing values for body mass index (BMI). After these exclusions, a total of 487,209 participants remained in the primary analysis. Reasons for exclusion were not mutually exclusive, with 1,406 participants meeting multiple exclusion criteria. For analysis of chronic respiratory diseases, participants with chronic obstructive pulmonary disease (COPD, n=37,057) or asthma (n=2,528) at baseline were further excluded, leaving 451,233 participants remaining in the analysis. Baseline COPD was ascertained based on self-reported clinical diagnosis of chronic bronchitis or emphysema and onsite pulmonary function test.^12^ Other medical histories relied on self-reported clinical diagnoses.

The CNHS (2015-2017) was the latest cross-sectional survey with nationally and provincially representative samples from 302 survey sites of 31 provincial-level administrative divisions in mainland China. In this round of surveillance, the survey on adult chronic diseases and nutritional status was conducted in 2015. Participants were sampled using a stratified multistage cluster sampling design, with details published previously.^13^ In the present study, we used up to 171,127 adults aged 30-84 years from the CNHS 2015 to estimate the sex- and age-specific (every 5-years) prevalence of lifestyle-related factors (Table S1). Ethical Committee of China CDC approved the survey. All participants had completed written informed consent forms.

### Data collection

Baseline lifestyle-related factors and covariate information in CKB were assessed by interviewer-administered laptop-based questionnaires (available at https://www.ckbiobank.org/) and physical measurements. The data entry system had built-in functions to avoid missing items and logic errors maximally. Details have been described in the Supplementary Material (appendix p2).

The data in CNHS were collected by face-to-face interviews with trained staff using well-designed questionnaires (appendix p3-7) and taking physical measurements. Questions about smoking status were basically the same as those in the CKB questionnaire, except that only the cigarettes were considered to calculate the daily smoking amount. A food frequency questionnaire was used to collect the frequency and amount of various foods and alcoholic drinks consumed in the past 12 months. Physical activity was investigated with an adapted version of the International Physical Activity Questionnaire (IPAQ)-long form, and total physical activity level was calculated in a manner similar to that in CKB (appendix p8). Body weight, height, and waist circumference (WC) were measured by trained staff using well-calibrated instruments.

The all-cause and cause-specific mortality rates, including from cardiovascular disease (CVD), cancer, and chronic respiratory disease (CRD) (including COPD and asthma), of the Chinese population by sex and 5-year age groups (30-94 years) in 2015 were derived from the GBD study (http://ghdx.healthdata.org/gbd-results-tool).

### Definition of low-risk lifestyle

Five modifiable lifestyle factors were included in this study based on previous studies and Dietary Guidelines for Chinese Residents:^1,5,7,14^ smoking, alcohol intake, physical activity, dietary habits, and body shape (a reflection of balance between energy intake and energy expenditure).

Not smoking or quitting smoking for reasons other than illness was defined as low risk. Former smokers who had stopped smoking due to illness were excluded from the low-risk group to avoid biasing death risk upward. The low-risk group for alcohol intake included non-regular drinkers and daily light-to-moderate drinkers (<30 g of pure alcohol in men and <15 g in women per day).^1,5^ Former drinkers were also excluded from the low-risk group to address the potential sick-quitter phenomenon.^15^ However, such exclusion did not apply to the CNHS because its questionnaire did not ask about previous drinking habits. The low-risk group for physical activity included those who engaged in an age- (<50, 50-59, and ≥60 years) and sex-specific median or higher level of physical activity.^1^ For dietary habits, we created a simple diet score by considering the following criteria: eating fresh vegetables daily, eating fresh fruits daily, eating red meat 1-6 days per week, eating legumes ≥4 days per week, and eating fish ≥1 day per week. For each criterion met, one point was scored; otherwise, 0. Thus, the diet score ranged from 0 to 5, with a score of 4 to 5 classified as the low-risk group.^1^ Both general and central adiposity indicators were considered for body shape, with BMI of 18.5 to 27.9 kg/m^2^ and WC of <90/85 cm for men/women defined as low-risk,^16^ which emphasizes prevention of extremely high or low weight and abdominal obesity.

A simple low-risk lifestyle score was derived based on the number of low-risk lifestyle factors, ranging from 0 to 5, with higher scores indicating a healthier lifestyle.

### Ascertainment of deaths

The vital status of each participant in CKB was identified through National Disease Surveillance Points (DSP) system, supplemented with the annual active follow-up. The underlying causes of death were coded using the 10th revision of the International Classification of Diseases (ICD-10). The primary outcomes were all-cause mortality and cause-specific mortality, including CVD [I00-I99], cancer [C00-C97], and CRD (including COPD [J41-J44] and asthma [J45-J46]).

### Statistical analysis

In the analysis of CKB, person years were counted from baseline until death, lost to follow-up, or December 31, 2017, whichever occurred first. Cox proportional hazards regression with an age timescale was used to calculate the hazard ratio (HR) and 95% confidence interval (CI) for the relative risk of mortality outcomes with each lifestyle factor and the number of combined lifestyle factors. The Cox model was stratified jointly by 10 study areas and age at baseline in a 5-year interval. For cause-specific mortality, we applied a regression model based on the proportional sub-distribution hazard proposed by Fine and Gray.^18^

Assuming that the observed association is causal, we calculated population-attributable risk percent (PAR%), which estimates the percentage of mortality that would have been prevented if all participants had been in the low-risk group. In these analyses, we coded low-risk lifestyle factors as a binary variable and compared participants with all five low-risk factors with all others, following a method advocated by Wacholder et al.^17^

The statistical methods used for estimating years of life gained or lost associated with lifestyle factors are detailed in the Supplementary Material (appendix p9-11). Due to the sex differences in life expectancy, we performed all analyses for men and women separately. We used period life tables to calculate the life expectancy, applying one-year age bands for age 30 through 94, with the final age group capturing those 95 years and over. The cumulative survival from the age of 30 onwards was estimated for participants following different levels of low-risk lifestyle factors by applying sex-specific HRs for all-cause and cause-specific mortality from the CKB to the detailed mortality component from the GBD, combined with the prevalence of low-risk lifestyle factors from CNHS (2015).

By applying Arriaga’s decomposition method,^18^ we estimated the cause-specific contributions to the life expectancy difference between participants adopting all five and 0-1 low-risk lifestyle factors to determine which cause-specific mortality differences were major contributors to the total change in life expectancy (detailed in appendix p12).

In the sensitivity analysis, we excluded CKB participants who died within the first two years of follow-up to minimize potential reverse causality. We also applied sex- and age-at-risk-specific HRs for all-cause mortality to the life expectancy analysis to account for potential non-linear increase of death hazard in older ages, in which participants’ age at risk was determined by splitting the follow-up time every 10 years old.^19^ The age-at-risk groups were set different for men (30-49, 50-59, 60-69, 70-79, 80- years) and women (30-69, 70- years), considering that few deaths occurred before the age of 70 and over 80 among women adopting 0-1 low-risk lifestyle factors (the reference group) in CKB study. Considering the lag time between exposure and mortality outcome, we substituted the mortality data with the most recent data from 2019 (4-year lag).^6,20^

Subgroup analyses were performed by the factors of residence (urban and rural), education level (illiterate and primary school, and middle school and above), smoking status (men: never, former, and current; women: never and ever), body shape (underweight, neither general nor abdominal obesity, and either or both), and baseline disease status (neither hypertension nor diabetes, and either or both).

Considering the gradients in death risk according to different levels of each lifestyle factor, we further created an expanded low-risk lifestyle score. We graded the categories of each lifestyle factor from 1 (least healthy) to 5 (most healthy) according to the CKB findings of the association between lifestyle factors and all-cause mortality. The points across all five lifestyle factors were summed, with the overall score ranging from 5 to 25.

All statistical analyses, unless otherwise stated, were performed using Stata (version 15.0, StataCorp). The competing-risk analysis, calculation of PAR%, and computation of prevalence of lifestyle factors were performed using SAS (version 9.4, SAS Institute Inc). The CI for life expectancy was estimated using @RISK 8.1 (Palisade Corp, Ithaca, NY).^21^ Graphs were plotted using R version 4.0.3.

### Role of the funding source

The funders had no role in the study design, data collection, data analysis and interpretation, writing of the report, or the decision to submit the article for publication.

## Results

The baseline mean age of included 487,209 CKB participants was 51.5 ± 10.5 years; 199,238 (40.9%) were men, and 277,062 (56.9%) resided in rural areas. A total of 331,104 (68.0%) participants had at least three low-risk lifestyle factors, 135,305 (27.8%) had at least four, and 9,767 (2.0%) had all five. Participants of women, younger age, being better educated, and urban residents were more likely to adopt a low-risk lifestyle (Table S2).

In CNHS, the mean age of the eligible 171,127 participants was 54.5 ± 12.3 years; 80,650 (47.1%) were men, and 101,707 (59.4%) resided in rural areas. Among all eligible participants, 46,559 (68.7%), 19,128 (28.2%), and 2,178 (3.2%) participants adopted at least three, four, and all five low-risk lifestyle factors. The subset of participants used for lifestyle combination analysis (n=67,798) shared similar characteristics with the whole participants.

During a median follow-up of 11.1 years (interquartile ranges 10.2-12.1; 5.3 million person-years), the CKB study documented 42,496 deaths, including 16,257 deaths from CVD, 14,069 deaths from cancer, and 3,332 deaths from CRD. After excluding participants with prevalent COPD and asthma at baseline, 1,449 deaths from CRD occurred among the remaining 451,233 participants. In the multivariable-adjusted model, smoking was associated with an increased risk of all-cause mortality; and both being physically active and following a healthy dietary habit were associated with a reduced risk of all-cause mortality (Table 1). Compared to alcohol drinkers who drank pure alcohol <15 g per day, former drinkers and heavy drinkers (>60 g/d) experienced a similarly higher death risk. We, therefore, combined these two groups for the subsequent analysis of life expectancy. Participants with a BMI of 18.5-27.9 kg/m^2^ but without abdominal obesity were at the lowest risk of all-cause mortality; both underweight and obesity, either general or abdominal, were associated with higher death risk. Most of the associations between individual lifestyle factors and mortality risks for CVD, cancer, and CRD were similar to those of all-cause mortality (Tables 1 and 2). Results by sex were shown in Tables S3 and S4.

**Figure 1:**
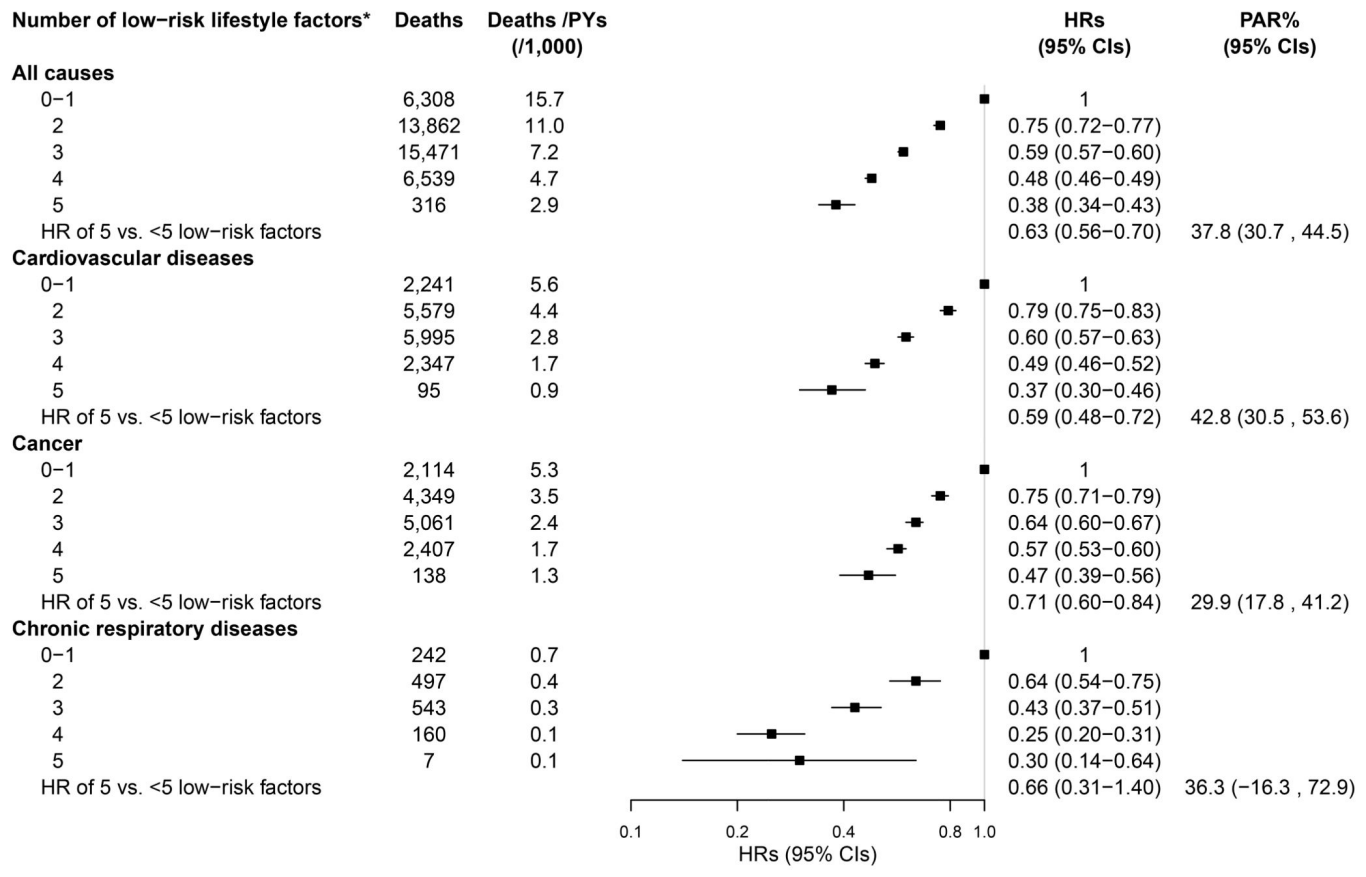
Multivariable-adjusted HRs (95% CIs) and PARs% (95% CIs) for all-cause and cause-specific mortality by the number of low-risk lifestyle factors. HR indicates hazard ratio; CI, confidence interval; PYs, person-years; PAR%, population attributable risk percent. For the analysis of death from chronic respiratory diseases, participants with chronic obstructive pulmonary disease (COPD; n=34,543) or asthma (n=2,528) at baseline were excluded. Multivariable models were adjusted for the same covariates as Table 1. *Low-risk lifestyle factors were defined as: never smoking or having stopped for reasons other than illness; less than daily drinking or drinking <30 g (men)/15 g (women) of pure alcohol per day (former drinkers excluded); engaging in an age- (<50 years, 50-59 years, and ≥60 years) and sex-specific median or higher level of physical activity; scoring 4 to 5 for all food groups; having a BMI between 18.5 and 27.9 kg/m^2^ and a waist circumference <90 cm (men)/85 cm (women).

**Figure 2:**
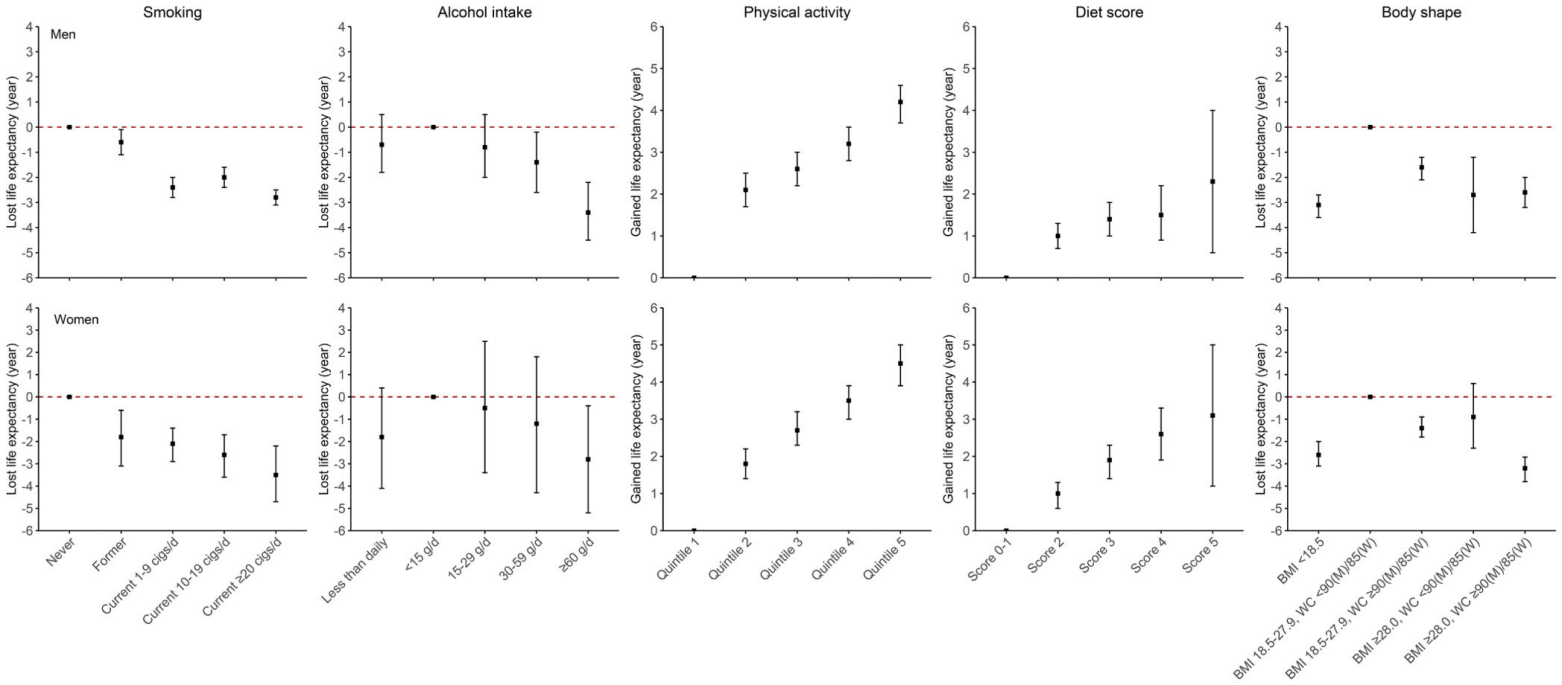
Projected gained or lost life expectancy at age 30 by individual lifestyle factors. cigs, cigarettes or equivalent; BMI, body mass index; WC, waist circumference; M, men; W, women. Former alcohol drinkers were included in the heavy drinking category (≥60 g of pure alcohol per day). The definition and classification of other lifestyle factors were the same as in Table 1.

When low-risk lifestyle factors were considered jointly, compared to participants with 0-1 low-risk lifestyle factor, the adjusted HR (95% CI) of participants who had five low-risk lifestyle factors was 0.38 (0.34, 0.43) for all-cause mortality, 0.37 (0.30, 0.46) for CVD mortality, 0.47 (0.39, 0.56) for cancer mortality, and 0.30 (0.14, 0.64) for CRD mortality. The PAR% (95%CI) of not adopting all five low-risk lifestyle factors was 37.8% (30.7%, 44.5%) for all-cause mortality, 42.8% (30.5%, 53.6%) for CVD mortality, 29.9% (17.8%, 41.2%) for cancer mortality, and 36.3% (-16.3%, 72.9%) for CRD mortality (Figure 1). The exclusion of deaths that occurred during the first two years of the follow-up did not substantially alter the results (Tables S5 and S6). Fine-Gray regression models yielded slightly attenuated risk estimates for cause-specific mortality (Table S7). All five low-risk lifestyle factors, including non-smoking, moderate alcohol intake, being physically active, healthy dietary habits, and absence of underweight or obesity, were associated with longer life expectancy (Figure 2).

**Figure 3:**
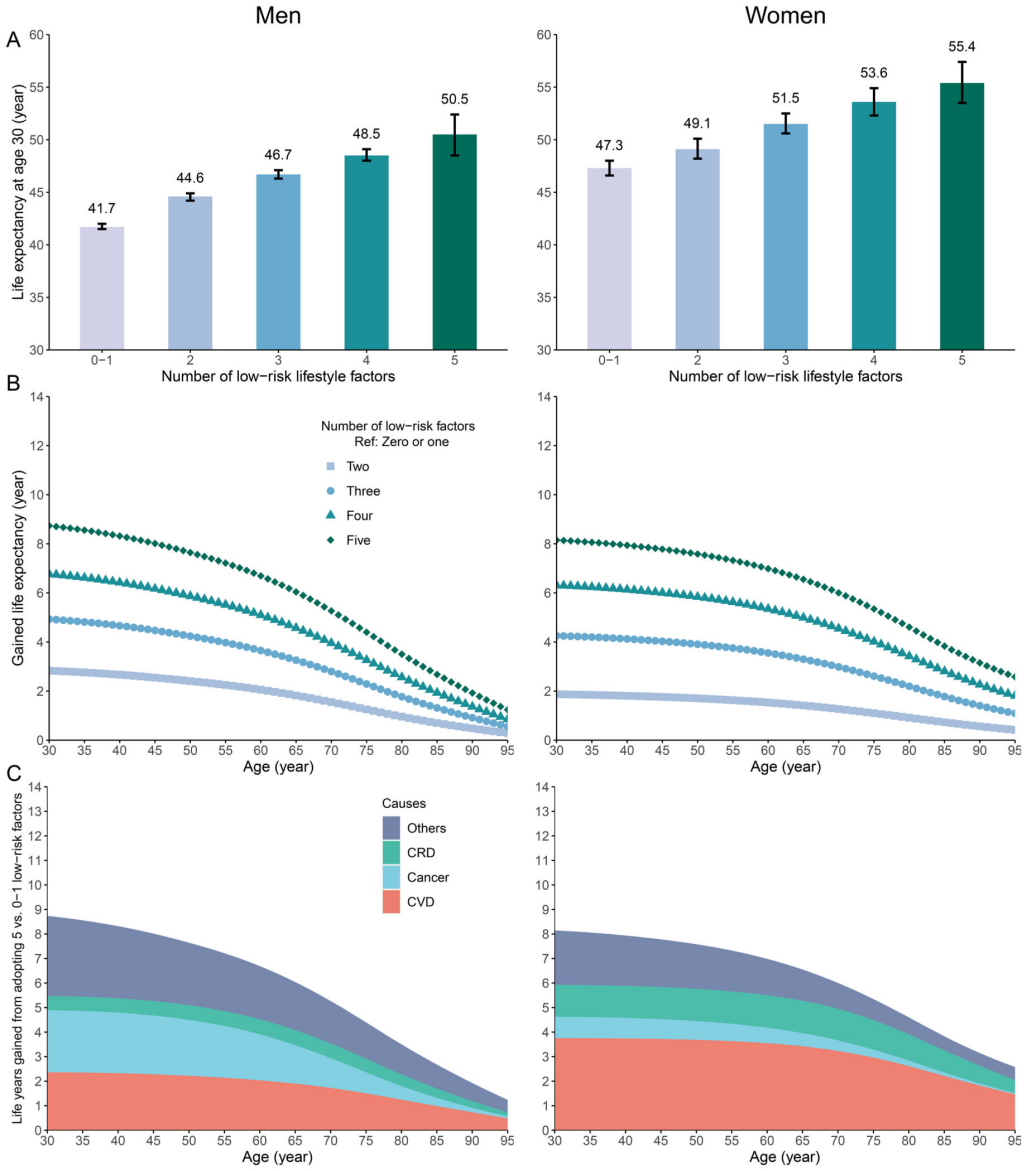
Life expectancy and years of life gained by the number of low-risk lifestyle factors and attribution of the causes of death. CVD, cardiovascular diseases; CRD, chronic respiratory diseases; Ref, reference. (A) Estimated life expectancy at age 30 by the number of low-risk lifestyle factors; (B) Gained life expectancy from adopting low-risk lifestyle; (C) Estimated years of life gained from adopting five versus 0-1 low-risk lifestyle factor attributable to reduced death from CVD, cancer, CRD, and other causes. The definition of low-risk lifestyle factors was the same as in Figure 1.

Using sex-specific HRs, the estimated life expectancy (95%CI) at age 30 for individuals with 0-1 low-risk lifestyle factor was 41.7 (41.5, 42.0) years for men and 47.3 (46.6, 48.0) years for women. For those who adopted all five low-risk lifestyle factors, the estimated life expectancy at age 30 was 50.5 (48.5, 52.4) years and 55.4 (53.5, 57.4) years for men and women, respectively (Figure 3A). By comparing individuals adopting all five with 0-1 low-risk lifestyle factors, the gain in life expectancy (95%CI) at age 30 years, on average, was 8.8 years (6.8, 10.7) for men and 8.1 years (6.5, 9.9) for women (Figure 3B; Table S8).

Compared to participants with 0-1 low-risk lifestyle factor, the gained years of life at age 30 from adopting five low-risk factors were attributable to reduced death from CVD (2.4 years [27% out of the total extended life expectancy] for men and 3.6 years [46%] for women), cancer (2.5 years [29%] and 0.9 years [11%]), CRD (0.6 years [7%] and 1.3 years [16%]), and other causes (3.3 years [37%] and 2.2 years [27%]) (Figure 3C). The life expectancy estimates remained robust in sensitivity analyses using sex- and age-at-risk specific HRs (Figure S3) and using mortality data from 2019 (Figure S4).

In subgroup analysis stratified by residence, education level, smoking status, obesity status, or disease status at baseline, we observed a consistent relationship between the increasing number of low-risk lifestyle factors and the gained life expectancy at age 30 years across subpopulations (Figures S5, S6, S7, S8, and S9). In the analysis using an expanded low-risk score, the average life expectancy at age 30 years for individuals scored ≥23 was 13.5 years and 12.1 years longer in men and women, respectively, compared to those scored ≤8 (Figure S10).

## Discussion

Our results suggest that adherence to each of the five low-risk lifestyle factors, namely never smoking or quitting for reasons other than illness, no excessive alcohol use, being physically active, healthy eating habits, having a BMI between 18.5 and 27.9 kg/m^2^ and without abdominal obesity, was associated with longer life expectancy for Chinese adults. The estimated life expectancy at age 30 for individuals with all five low-risk factors was on average 8.8 years longer in men and 8.1 years longer in women than those with 0-1 low-risk factors. The estimated improved life expectancy for men and women was mostly attributable to reduced death from CVD, cancer, and CRD.

To the best of our knowledge, this is the first study to quantify the association between combined lifestyle factors and life expectancy in China. In 2015, the average life expectancy at age 30 for Chinese adults was 45.5 years for men and 51.3 years for women.^22^ In the present study, the estimated life expectancy at age 30 for individuals with 0-1 low-risk lifestyle factors was 41.7 years for men and 47.3 years for women. However, adopting all five low-risk lifestyle factors was associated with an improved life expectancy at age 30, reaching 50.5 years for men and 55.4 years for women. Findings from the Singapore Chinese Health Study (SCHS) of a median of 20.6 years of follow-up data showed that the differences in life expectancy at age 50 were 6.6 years for men and 8.1 for women by comparing individuals with 4-5 versus zero low-risk lifestyle factors.^23^ In the present study, the corresponding estimates of gained life years at 50 years were 7.7 (6.0, 9.5) years for men and 7.6 (6.0, 9.3) years for women (Table S8), similar to the estimates from the study above but with smaller sex difference.

Our findings were consistent with previous studies in developed countries; that is, life expectancy increased with increasing numbers of low-risk lifestyle factors. Adherence to a healthy lifestyle was associated with a 17.9 years increase in life expectancy at age 20 for Canadians,^6^ and 12.2 years (for men) and 14 years (for women) at age 50 for Americans,^5^ and 18.5 years (for men) and 15.7 years (for women) at age 40 for EPIC-Heidelberg cohort population from Germany.^7^ In contrast, the estimates of gained life years in our study were lower than that of the above studies. Such inconsistency might be explained by the differences between populations in the definitions and components of a healthy lifestyle and their prevalence.^5^ Also, in developing countries, potential environmental hazards in the home, work, and broader outdoor environment, such as ambient and household air pollution, and chemical contamination of food and water, may lead to a significant burden of diseases.^24^ Hence, the relative impact of a healthy lifestyle alone on life expectancy might be slightly diminished in developing countries.

In cause-specific decomposition analysis of the life expectancy differences, we observed that compared to individuals with 0-1 low-risk lifestyle factors, about two-thirds of the increased life expectancy from adopting all five low-risk factors could be explained by the reduced death from CVD, cancer, and CRD, all representing the leading causes of death in Chinese population. Nevertheless, sex differences exist in the contribution percentages, with women (73%) higher than men (63%). In addition, the major contributors to the life expectancy difference were from CVD and CRD among women and from cancer and other causes among men. This difference might be related to the sex differences in the relative risks of lifestyle risk factors for various outcomes, disease burden patterns, and prevalence of lifestyle risk factors.

The lifestyle-related factors included in this study and the definition of their low-risk group were basically consistent with previous studies, except for physical activity and obesity indicators. Many studies in Western populations specifically focused on leisure-time physical activity. However, most of the physical activity in the current population was occupational (62%) and household (26%).^25^ We, therefore, defined the low-risk group based on total physical activity, and being physically active was associated with an increase in life expectancy at age 30 by more than four years. Despite the lack of comparison to findings from Western populations, our definition of physical activity is more meaningful to the Chinese population. As to adiposity measures, different from previous studies that only included BMI,^5,7,23^ this study used both BMI and WC. A recent meta-analysis of 72 prospective studies has suggested that the measures of central adiposity could be used with BMI as an auxiliary indicator to determine the risk of premature death.^26^

This study has several strengths. First, the nature of the CKB study in terms of its large sample size, long-term follow-up, and a large number of documented deaths enables us to obtain more precise sex-specific effect estimates for all-cause and cause-specific mortality than smaller studies. The inclusion of a geographically spread population living in urban and rural areas, with different socio-demographic characteristics, and the loss to follow-up rate of <1%, make the effect estimates broadly applicable. Second, we used a nationally representative survey to estimate the prevalence of lifestyle factors, improving the representativeness of the findings for the Chinese population. Third, existing studies mainly investigated the impact of lifestyle factors on the life expectancy at middle and old age, such as life expectancy at 50 years.^5,23^ The present study expands on prior findings and supports the benefits of starting a healthy lifestyle early at a young age, like 30 years.

Several limitations also merit discussion when interpreting the results. First, the lifestyle behaviors were self-reported in CKB and CNHS, most likely leading to biased results towards the null in the estimated associations and overestimated the prevalence of low-risk lifestyle factors. Second, we only used information on lifestyle factors at one time-point at baseline in the CKB without considering their potential changes during the follow-up. However, one of our previous studies using resurvey data from a subset of the CKB population showed that the lifestyle of most participants remained relatively stable over long periods.^27^ Third, we dichotomized each lifestyle factor and simply counted the number of low-risk lifestyle factors, ignoring the difference in the magnitude of association between various lifestyle factors and death. However, two prior studies compared the analyses using weighted lifestyle scores with non-weighted scores, and no significant differences were observed.^23^ Fourth, the definitions of low-risk lifestyle factors might not be entirely consistent between the CKB and CNHS due to subtle differences in the questionnaires. Nevertheless, slight changes in the prevalence of lifestyle factors would not substantially impact the results of our study under different simulation scenarios. Other limitations include the observational nature of the study precluding causal inference, not fully representativeness of the Chinese population for the CKB cohort, and the neglect of potential secular changes in health risk factors or clinical advance.

The present study of the Chinese population shows that adopting a low-risk lifestyle was associated with an increase in life expectancy at age 30 by 8.8 and 8.1 years in men and women by reducing the death from CVD, cancer, and CRD. Assuming that the observed associations are causal, there is still much room for improvement in the life expectancy of the Chinese population through population-wide healthy lifestyles interventions. A latest study from Hong Kong of China has shown the possibility of realizing this vision, emphasizing the critical role of tobacco control in improving life expectancy.^28^

## Supplementary Material

Supplementary file

## Figures and Tables

**Table 1:** Multivariable-adjusted hazard ratios (95% CIs) for all-cause and cause-specific mortality by individual lifestyle risk factors among 487,209 participants

	All causes				Cardiovascular diseases				Cancer		
	Deaths	Deaths/PYs (/1,000)	HR (95% CI)		Deaths	Deaths/PYs (/1,000)	HR (95% CI)		Deaths	Deaths/PYs (/1,000)	HR (95% CI)
**Smoking** *											
Never	21,819	6.0	1.00 (Referent)		8,899	2.5	1.00 (Referent)		6,838	1.9	1.00 (Referent)
Former	1,929	12.8	1.11 (1.06-1.17)		707	4.7	1.04 (0.96-1.13)		655	4.4	1.15 (1.06-1.26)
Current (cigarettes or equivalents/day)									
1-9	4,832	16.5	1.30 (1.25-1.35)		2,010	6.9	1.27 (1.20-1.35)		1,411	4.8	1.34 (1.25-1.43)
10-19	4,968	12.1	1.26 (1.21-1.31)		1,797	4.4	1.20 (1.13-1.27)		1,699	4.1	1.39 (1.30-1.48)
≥20	8,948	10.9	1.38 (1.34-1.43)		2,844	3.5	1.28 (1.21-1.35)		3,466	4.2	1.59 (1.50-1.69)
**Alcohol intake** †											
Less than daily	33,334	7.2	1.08 (0.97-1.21)		13,331	2.9	1.12 (0.93-1.34)		10,559	2.3	0.96 (0.80-1.15)
Former	3,410	18.0	1.42 (1.27-1.59)		1,185	6.3	1.37 (1.14-1.65)		1,067	5.6	1.22 (1.01-1.47)
Current daily (g of pure alcohol/day)									
<15	337	13.3	1.00 (Referent)		123	4.9	1.00 (Referent)		122	4.8	1.00 (Referent)
15-29	1,073	11.7	1.11 (0.98-1.25)		377	4.1	1.14 (0.93-1.40)		425	4.6	1.09 (0.89-1.33)
30-59	1,791	11.4	1.18 (1.05-1.33)		558	3.6	1.17 (0.96-1.42)		740	4.7	1.14 (0.94-1.38)
≥60	2,551	12.4	1.41 (1.26-1.58)		683	3.3	1.35 (1.11-1.64)		1,156	5.6	1.48 (1.23-1.79)
**Physical activity** ‡											
Quintile 1	10,906	10.4	1.00 (Referent)		4,556	4.3	1.00 (Referent)		3,188	3.0	1.00 (Referent)
Quintile 2	8,732	8.3	0.82 (0.80-0.84)		3,506	3.3	0.79 (0.76-0.83)		2,789	2.6	0.92 (0.87-0.97)
Quintile 3	8,097	7.7	0.76 (0.74-0.78)		3,008	2.8	0.70 (0.67-0.74)		2,770	2.6	0.91 (0.86-0.96)
Quintile 4	7,795	7.3	0.71 (0.69-0.73)		2,803	2.6	0.67 (0.63-0.70)		2,735	2.6	0.88 (0.83-0.92)
Quintile 5	6,966	6.5	0.65 (0.63-0.67)		2,384	2.2	0.61 (0.58-0.64)		2,587	2.4	0.81 (0.77-0.86)
**Diet score** §											
0-1	11,994	10.6	1.00 (Referent)		5,352	4.7	1.00 (Referent)		3,332	2.9	1.00 (Referent)
2	17,823	8.3	0.90 (0.88-0.92)		6,499	3.0	0.88 (0.84-0.91)		5,901	2.7	0.95 (0.91-0.99)
3	10,297	6.5	0.84 (0.81-0.87)		3,628	2.3	0.81 (0.77-0.85)		3,841	2.4	0.92 (0.87-0.98)
4	2,163	5.6	0.80 (0.76-0.84)		711	1.8	0.73 (0.67-0.79)		907	2.3	0.92 (0.85-1.00)
5	219	5.4	0.74 (0.65-0.85)		67	1.6	0.62 (0.49-0.80)		88	2.2	0.80 (0.64-0.99)
Body shape											
BMI <18.5	4,282	19.5	1.37 (1.32-1.42)		1,439	6.6	1.13 (1.07-1.20)		1,055	4.8	1.29 (1.20-1.38)
BMI 18.5-, WC <90(M)/85(W)	27,744	7.3	1.00 (Referent)		10,155	2.7	1.00 (Referent)		9,708	2.6	1.00 (Referent)
BMI 18.5-, WC ≥90(M)/85(W)	6,523	8.8	1.20 (1.16-1.23)		2,870	3.9	1.30 (1.24-1.37)		2,042	2.8	0.99 (0.94-1.05)
BMI 28.0-, WC <90(M)/85(W)	310	5.2	1.21 (1.08-1.36)		129	2.2	1.47 (1.23-1.75)		110	1.8	1.00 (0.82-1.20)
BMI 28.0-, WC ≥90(M)/85(W)	3,637	7.5	1.40 (1.34-1.47)		1,664	3.4	1.66 (1.55-1.78)		1,154	2.4	1.01 (0.94-1.09)

BMI indicates body mass index; WC, waist circumference; PYs, person-years; HR, hazard ratio; CI, confidence interval; M, men; W, women. Multivariable models were adjusted for sex (men or women), education (no formal school, primary school, middle school, high school, college, or university or higher), marital status (married, widowed, divorced or separated, or never married), hip circumference (mm), family histories of heart attack and stroke (presence, absence, or unknown; adjusted for analyses of all-cause and cardiovascular mortality), and family history of cancer (presence, absence, or unknown; adjusted for analyses of all-cause and cancer mortality). All five lifestyle factors were included simultaneously in the same model.

* Former smokers referred to those having stopped smoking for reasons other than illness. Participants who had stopped smoking due to illness were classified as current smokers.

† Less than daily group included never-regular drinkers and current weekly drinkers. Former drinkers referred to those who used to drink at least once weekly but drank less than weekly at baseline.

‡ Physical activity level was categorized based on age- (<50 years, 50-59 years, and ≥60 years) and sex-specific quintile of total physical activity level.

§ Diet score was created based on the following criteria: eating fresh vegetables daily, eating fresh fruits daily, eating red meat 1-6 days per week, eating legumes ≥4 days per week, eating fish ≥1 day per week. For each food group, the participant who met the criterion received a score of 1, and otherwise, 0.

**Table 2:** Multivariable-adjusted hazard ratios (95% CIs) for mortality of chronic respiratory diseases by individual lifestyle risk factors among 451,233 participants*

	Deaths	Deaths/PYs (/1,000)	HR (95% CI)
**Smoking**			
Never	664	0.2	1.00 (Referent)
Former	59	0.4	1.29 (0.97-1.72)
Current (cigarettes or equivalents/day)			
1-9	247	0.9	1.78 (1.49-2.13)
10-19	209	0.6	1.53 (1.26-1.86)
≥20	270	0.4	1.59 (1.32-1.93)
Alcohol intake			
Less than daily	1,149	0.3	0.85 (0.50-1.45)
Former	123	0.8	1.10 (0.63-1.91)
Current daily (g of pure alcohol/day)			
<15	14	0.6	1.00 (Referent)
15-29	34	0.4	0.82 (0.44-1.53)
30-59	52	0.4	0.83 (0.46-1.50)
≥60	77	0.4	0.96 (0.54-1.72)
**Physical activity**			
Quintile 1	322	0.3	1.00 (Referent)
Quintile 2	309	0.3	0.88 (0.75-1.03)
Quintile 3	253	0.3	0.70 (0.59-0.83)
Quintile 4	291	0.3	0.64 (0.54-0.75)
Quintile 5	274	0.3	0.55 (0.46-0.66)
**Diet score**			
0-1	531	0.5	1.00 (Referent)
2	584	0.3	0.79 (0.69-0.91)
3	286	0.2	0.77 (0.64-0.91)
4	45	0.1	0.68 (0.49-0.94)
5	3	0.1	0.49 (0.16-1.54)
Body shape			
BMI <18.5	329	1.8	2.15 (1.86-2.50)
BMI 18.5-, WC <90(M)/85(W)	855	0.2	1.00 (Referent)
BMI 18.5-, WC ≥90(M)/85(W)	184	0.3	1.45 (1.21-1.74)
BMI 28.0-, WC <90(M)/85(W)	7	0.1	1.90 (0.90-4.02)
BMI 28.0-, WC ≥90(M)/85(W)	74	0.2	1.86 (1.40-2.47)

BMI indicates body mass index; WC, waist circumference; PYs, person-years; HR, hazard ratio; CI, confidence interval; M, men; W, women. The definition and classification of individual lifestyle factors were the same as in Table 1. Multivariable model was adjusted for sex, education, marital status, and hip circumference at baseline. All five lifestyle factors were included simultaneously in the same model.

* Participants with chronic obstructive pulmonary disease (COPD; n=34,543) or asthma (n=2,528) at baseline were excluded from the analysis population in Table 1.

## Data Availability

Details of how to access China Kadoorie Biobank data and details of the data release schedule are available from www.ckbiobank.org/site/Data+Access. The CNHS data will be available from the corresponding authors on request.

## Research in context

### Evidence before this study

We searched PubMed, EMBASE, and Google Scholar for articles published from the inception of each database to October 31, 2021, using a combination of terms: (“life expectancy” OR “life span” OR “life time” OR “life years” OR “longevity”) AND (“lifestyle” OR “smoking” OR “tobacco use” OR “alcohol” OR “physical activity” OR “diet” OR “BMI” OR “overweight” OR “obesity”). We implemented no restriction on study type or language. Relevant studies were also found by checking reference lists of identified articles. Available studies that assessed the relationship between lifestyle and life expectancy were mainly performed in Western populations and based on specific cohort populations, limiting the generalizability of the results. It remains unclear how much the impact of population-wide healthy lifestyles initiative will have on the life expectancy of populations from countries like China, where the factors that influence health are complex and varied.

### Added value of this study

The estimated life expectancy at age 30 years for individuals with all five low-risk factors was on average 8.8 years longer in men and 8.1 years longer in women than those with 0-1 low-risk factors. About two-thirds of the extended life expectancy from adopting all five low-risk factors could be explained by the reduced death from cardiovascular disease, cancer, and chronic respiratory disease. To the best of our knowledge, this is the first study to quantify the association between combined lifestyle factors and life expectancy in China. Using a large prospective cohort study of 0.5 million Chinese and a nationally representative survey of risk factors improves the representativeness of the findings for the Chinese population.

### Implications of all the available evidence

Our findings suggest that fostering a healthy lifestyle through population-wide public health interventions could be associated with substantial gains in life expectancy of the Chinese population. The findings of the study may help the government commit to promoting a healthy lifestyle to achieve the goal of increasing the average life expectancy as outlined in the blueprint of Healthy China 2030. Further researches are also needed to explore the impact of other factors on life expectancy, such as environmental hazards.
