# Supplementary material for: Healthy lifestyle and life expectancy at age 30 in the Chinese population: an observational study
Source: Lancet Public Health. Author manuscript; Available in PMC 2023 Aug 31. (PMC7615002; doi:10.1016/S2468-2667(22)00110-4)
Supplement: Supplementary file [file EMS185043-supplement-Supplementary_file.pdf]

THE LANCET
Public Health

Supplementary appendix

This appendix formed part of the original submission and has been peer reviewed. We post it as supplied by the authors.

Supplement to: Sun Q, Yu D, Fan J, et al. Healthy lifestyle and life expectancy at age 30 years in the Chinese population: an observational study. *Lancet Public Health* 2022; published online Aug 1. https://doi.org/10.1016/S2468-2667(22)00110-4.

Supplementary Material

Members of the China Kadoorie Biobank collaborative group 1
Baseline survey of the China Kadoorie Biobank (CKB) study 2
Questionnaire used in the China Nutrition and Health Surveillance (CNHS) 3
Section 1: Smoking 3
Section 2: Alcohol drinking 4
Section 3: Consumption of legumes, meat, fish, fruits, and vegetables 5
Section 4: Physical activity 6
Physical activity domains, types, intensity categories, and MET values in CNHS 8
Statistical method used for estimating years of life gained or lost 9
Statistical method used for estimating contributions of specific causes of death to the absolute difference in life expectancy 11
References 12
Table S1. Numbers of the CNHS participants used to estimate the prevalence of individual or combined lifestyle factors* 13
Table S2. Baseline characteristics of the CKB study participants according to number of low-risk lifestyle factors 14
Table S3. Multivariable-adjusted hazard ratios (95% CIs) for all-cause and cause-specific mortality among men in CKB study 16
Table S4. Multivariable-adjusted hazard ratios (95% CIs) for all-cause and cause-specific mortality among women in CKB study 18
Table S5. Sensitivity analyses for all-cause and cause-specific mortality by excluding deaths during the first two years of follow-up in CKB study 20
Table S6. Sensitivity analyses for mortality of chronic respiratory diseases by excluding deaths during the first two years of follow-up in CKB study* 22
Table S7. Competing risk analyses for cause-specific mortality by using Fine and Gray regression model 24
Table S8. Years of life gained at age 30, 50, and 65 years by the number of low-risk lifestyle factors. 26
Figure S1. Data sources and steps used to calculate the life expectancy of each lifestyle factor group. 27
Figure S2. Observed and predicted all-cause and cause-specific mortality rates of Chinese population in 2015. 28
Figure S3. Sensitivity analyses of estimated life expectancy at age 30 according to the number of low-risk lifestyle factors by using sex- and age-at-risk specific hazard ratios. 29
Figure S4. Sensitivity analyses of estimated life expectancy and years of life gained by the number of low-risk lifestyle factors and attribution of the causes of death using mortality data from 2019. 30
Figure S5. Estimated life expectancy at age 30 according to the number of low-risk factors stratified by residence. 31
Figure S6. Estimated life expectancy at age 30 according to the number of low-risk factors stratified by education level. 32
Figure S7. Estimated life expectancy at age 30 according to the number of low-risk factors stratified by smoking status. 33
Figure S8. Estimated life expectancy at age 30 according to the number of low-risk factors stratified by obesity status. 34

Figure S9. Estimated life expectancy at age 30 according to the number of low-risk factors stratified by baseline status of hypertension and diabetes. 35
Figure S10. Hazard ratios for all-cause mortality and estimated life expectancy according to expanded low-risk score. 36

Members of the China Kadoorie Biobank collaborative group

International Steering Committee: Junshi Chen, Zhengming Chen (PI), Robert Clarke, Rory Collins, Yu Guo, Liming Li (PI), Jun Lv, Richard Peto, Robin Walters. **International Co-ordinating Centre, Oxford:** Daniel Avery, Ruth Boxall, Derrick Bennett, Yumei Chang, Yiping Chen, Zhengming Chen, Robert Clarke, Huaidong Du, Simon Gilbert, Alex Hacker, Mike Hill, Michael Holmes, Andri Iona, Christiana Kartsonaki, Rene Kerosi, Ling Kong, Om Kurmi, Garry Lancaster, Sarah Lewington, Kuang Lin, John McDonnell, Iona Millwood, Qunhua Nie, Jayakrishnan Radhakrishnan, Paul Ryder, Sam Sansome, Dan Schmidt, Paul Sherliker, Rajani Sohoni, Becky Stevens, Iain Turnbull, Robin Walters, Jenny Wang, Lin Wang, Neil Wright, Ling Yang, Xiaoming Yang. **National Co-ordinating Centre, Beijing:** Yu Guo, Xiao Han, Can Hou, Jun Lv, Pei Pei, Chao Liu, Canqing Yu. **10 Regional Co-ordinating Centres: Qingdao CDC:** Zengchang Pang, Ruqin Gao, Shanpeng Li, Shaojie Wang, Yongmei Liu, Ranran Du, Yajing Zang, Liang Cheng, Xiaocao Tian, Hua Zhang, Yaoming Zhai, Feng Ning, Xiaohui Sun, Feifei Li. **Licang CDC:** Silu Lv, Junzheng Wang, Wei Hou. **Heilongjiang Provincial CDC:** Mingyuan Zeng, Ge Jiang, Xue Zhou. **Nangang CDC:** Liqiu Yang, Hui He, Bo Yu, Yanjie Li, Qinai Xu,Quan Kang, Ziyan Guo. **Hainan Provincial CDC:** Dan Wang, Ximin Hu, Jinyan Chen, Yan Fu, Zhenwang Fu, Xiaohuan Wang. **Meilan CDC:** Min Weng, Zhendong Guo, Shukuan Wu,Yilei Li, Huimei Li, Zhifang Fu. **Jiangsu Provincial CDC:** Ming Wu, Yonglin Zhou, Jinyi Zhou, Ran Tao, Jie Yang, Jian Su. **Suzhou CDC:** Fang liu, Jun Zhang, Yihe Hu, Yan Lu, , Liangcai Ma, Aiyu Tang, Shuo Zhang, Jianrong Jin, Jingchao Liu. **Guangxi Provincial CDC:** Zhenzhu Tang, Naying Chen, Ying Huang. **Liuzhou CDC:** Mingqiang Li, Jinhuai Meng, Rong Pan, Qilian Jiang, Jian Lan,Yun Liu, Liuping Wei, Liyuan Zhou, Ningyu Chen Ping Wang, Fanwen Meng, Yulu Qin,, Sisi Wang. **Sichuan Provincial CDC:** Xianping Wu, Ningmei Zhang, Xiaofang Chen,Weiwei Zhou. **Pengzhou CDC:** Guojin Luo, Jianguo Li, Xiaofang Chen, Xunfu Zhong, Jiaqiu Liu, Qiang Sun. **Gansu Provincial CDC:** Pengfei Ge, Xiaolan Ren, Caixia Dong. **Maiji CDC:** Hui Zhang, Enke Mao, Xiaoping Wang, Tao Wang, Xi zhang. **Henan Provincial CDC:** Ding Zhang, Gang Zhou, Shixian Feng, Liang Chang, Lei Fan. **Huixian CDC:** Yulian Gao, Tianyou He, Huarong Sun, Pan He, Chen Hu, Xukui Zhang, Huifang Wu, Pan He. **Zhejiang Provincial CDC:** Min Yu, Ruying Hu, Hao Wang. Tongxiang CDC: Yijian Qian, Chunmei Wang, Kaixu Xie, Lingli Chen, Yidan Zhang, Dongxia Pan, Qijun Gu. **Hunan Provincial CDC:** Yuelong Huang, Biyun Chen, Li Yin, Huilin Liu, Zhongxi Fu, Qiaohua Xu. **Liuyang CDC:** Xin Xu, Hao Zhang, Huajun Long, Xianzhi Li, Libo Zhang, Zhe Qiu.

Baseline survey of the China Kadoorie Biobank (CKB) study

Assessment of lifestyle factors

In the baseline questionnaire of the CKB study, ever smokers were asked for frequency, type, and the amount of tobacco smoked per day, and former smokers were also asked for years since stopping and the reason for quitting smoking. Participants who drank at least once a week were asked for the types of alcoholic beverages consumed and the amount drunk on a typical drinking day in the prior 12 months. For physical activity, participants were asked about the usual type and duration of activities in occupational, commuting, domestic, and leisure-time related domains in the past 12 months. Total physical activity was calculated by multiplying the metabolic equivalent of task (MET) value of each type of activity and the hours spent on that activity per day and then summarizing the MET-hours for all activities.[1] Qualitative habitual intakes of 12 conventional food groups in the past 12 months were assessed using a validated food frequency questionnaire.[2] Trained staff used calibrated instruments to measure weight, height, and waist and hip circumferences. Body mass index (BMI) was defined as weight in kilograms divided by height in meters squared.

Assessment of covariates

Covariate information included sociodemographic characteristics, personal and family medical history, and women's menopausal status. A participant was considered as having a family history of a particular disease if he reported to have one or more first-degree relatives with that disease. Prevalent hypertension was defined as measured systolic blood pressure ≥140 mmHg, measured diastolic blood pressure ≥90 mmHg, self-reported diagnosis of hypertension, or self-reported use of antihypertensive medication at baseline. Prevalent diabetes was defined as self-reported diabetes or screen-detected diabetes. The screen-detected diabetes was defined as measured fasting blood glucose >7.0 mmol/L or random blood glucose >11.1 mmol/L at baseline.

Questionnaire used in the China Nutrition and Health Surveillance (CNHS)

Section 1: Smoking

1. Do you now smoke every day, some days, or not at all?
   - □ Now smoke every day → ***go to Question 3***
   - □ Now smoke some days
   - □ Have smoked, do not smoke at all now
   - □ Never smoke → ***go to the end***
2. In the past, have you ever smoked every day?
   - □ Yes
   - □ No
3. At about what age did you first start smoking every day? __Years
4. How many machine-rolled cigarettes do you smoke per day (per week) on average now?
   - □ __number/day
   - □ __number/week
   - □ Other tobacco products
5. How many years ago did you last stop smoking regularly?
   - □ ___Years
   - □ ___Months
   - □ ___Weeks
   - □ ___Days
6. What was your main reason for stopping smoking?
   - □ Physical illness that you already had
   - □ Health concerns (about future illness)
   - □ Money
   - □ Family against
   - □ Advice from the doctor
   - □ Other

Section 2: Alcohol drinking

Please recall whether you have drunk any of the following alcoholic beverages on a regular basis in the past 12 months and estimate the drinking frequency and quantity.

	Drink or not	**Frequency (fill in only one of them)**				Average Intake/Time
		Times/ Day	Times/ Week	Times/ Month	Times/ Year	
Spirit (≥42% alcohol)	□	□	□	□	□	__g
Spirit (<42% alcohol)	□	□	□	□	□	__g
Beer (4% alcohol)	□	□	□	□	□	__ml
Huangjiu (18% alcohol)	□	□	□	□	□	__g
Rice wine (18% alcohol)	□	□	□	□	□	__g
Grape wine (14% alcohol)	□	□	□	□	□	__g

Section 3: Consumption of legumes, meat, fish, fruits, and vegetables

Please recall whether you ate the following food items in the past 12 months or not, and estimate the frequency and average edible amount.

	Eat or not	Frequency (fill in only one of them)				Average intake/Time
		Times/ Day	Times/ Week	Times/ Month	Times/ Year	
Soybean products						
Soybeans	□	□	□	□	□	__g
Soy milk/tofu jelly	□	□	□	□	□	__g
Tofu	□	□	□	□	□	__g
Yuba	□	□	□	□	□	__g
Wensi bean curd	□	□	□	□	□	__g
Meat						
Pork	□	□	□	□	□	__g
Beef	□	□	□	□	□	__g
Mutton	□	□	□	□	□	__g
Poultry meat	□	□	□	□	□	__g
Other meat (rabbit etc.)	□	□	□	□	□	__g
Meat product (sausage etc.)	□	□	□	□	□	__g
Edible offal	□	□	□	□	□	__g
Fish/sea food						
Sea fish	□	□	□	□	□	__g
Freshwater fish	□	□	□	□	□	__g
Shrimp	□	□	□	□	□	__g
Crab	□	□	□	□	□	__g
Mollusca (squid etc.)	□	□	□	□	□	__g
Fruits*						
Fresh fruits	□	□	□	□	□	__g
Dried fruits	□	□	□	□	□	__g
Vegetables*						
Fresh vegetables	□	□	□	□	□	__g
Dried vegetables	□	□	□	□	□	__g
Pickled vegetables	□	□	□	□	□	__g

*For consistency with the CKB questionnaire, we only counted the intake frequency of fresh vegetables and fruits.

Section 4: Physical activity

The following questions are based on your physical activity (including farming, occupation, domestic, commuting, and leisure-time activity) in a typical week.

- **Occupation, farming, and domestic activity**

1. Do you do vigorous physical activities that you did for at least 10 minutes at a time as part of your occupation, farming, or domestic activity?
   (vigorous physical activities refer to activities that take hard physical effort and make you breathe much harder than normal or fasten up your heart rate, such as heavy lifting, digging)
   □ Yes
   □ No → ***go to Question 4***
2. In a typical week, on how many days do you do vigorous physical activities as part of your occupation, farming, or domestic activity? __Days
3. How much time do you usually spend on a typical day doing vigorous physical activities as part of your occupation, farming, or domestic activity?
   __hours__minutes
4. Do you do moderate physical activities that you did for at least 10 minutes at a time as part of your occupation, farming work, or domestic activity?
   (moderate activities refer to activities that take moderate physical effort and make you breathe somewhat harder than normal or slightly fasten up your heart rate, such as sawing, laundry, cleaning)
   □ Yes
   □ No → ***go to Question 7***
5. In a typical week, on how many days do you do moderate physical activities as part of your occupation, farming, or domestic activity? __Days
6. How much time do you usually spend on a typical day doing moderate physical activities as part of your occupation, farming, or domestic activity?
   __hours__minutes

- **Commuting activity**

The following questions do not include occupation, farming, and domestic activity mentioned above.

7. Have you ever walked or bicycled for at least 10 minutes while out and about?
   □ Yes
   □ No → ***go to Question 10***
8. In a typical week, on how many days do you walk or bicycle for at least 10 minutes while out and about? __Days
9. How much time do you usually spend on a typical day walking or bicycling?
   __hours__minutes

- **Leisure time activity**

The following questions do not include occupation, farming, domestic and commuting activity mentioned above.

10. Do you do vigorous physical activities like long-distance running, swimming, and playing football that you did for at least 10 minutes at a time in your leisure time?
    □ Yes

□ No → ***go to Question 13***

11. In a typical week, on how many days do you do vigorous physical activities in your leisure time?
Days
12. How much time do you usually spend on a typical day doing vigorous physical activities in your leisure time?
__hours__minutes
13. Do you do moderate physical activities like brisk walking and Tai Chi that you did for at least 10 minutes at a time in your leisure time?
□ Yes
□ No → ***go to the end***
14. In a typical week, on how many days do you do moderate physical activities in your leisure time?
Days
15. How much time do you usually spend on a typical day doing moderate physical activities in your leisure time?
__hours__minutes

Physical activity domains, types, intensity categories, and MET values in CNHS

Domain	Activity type	Intensity	MET value*
Occupational/domestic activity	Moderate activity	Moderate	4.0
	Vigorous activity	Vigorous	6.8
Commuting activity	Bicycling/walking	Moderate	4.7
Leisure-time activity	Moderate activity	Moderate	4.0
	Vigorous activity	Vigorous	8.0

MET, metabolic equivalent of task.

*The MET value for each activity type was assigned based on the International Physical Activity Questionnaire (IPAQ) criteria.[3]

Statistical method used for estimating years of life gained or lost

We combined three pieces of information to estimate the changes in life expectancy associated with adopting different levels of individual or combined lifestyle factors:

(1) hazard ratios (HRs) for all-cause and cause-specific mortality associated with low-risk lifestyle factors derived from the CKB;
(2) population-based all-cause and cause-specific mortality rates from the GBD 2015;
(3) population-based prevalences of low-risk lifestyle factors estimated from the CNHS 2015.

We built life tables for individual and combined low-risk lifestyle factors in men and women separately, based on the estimated sex- and age-specific mortality rates in each lifestyle factor category. The sex- and age-specific mortality rates for each lifestyle factor category were estimated using sex-specific HRs for mortality from the CKB, the overall population mortality rates by sex and 5-year age intervals from the GBD, and the prevalence of low-risk lifestyle factors by sex and 5-year age intervals from the CNHS. The detailed information is described below.

Sex-specific HRs for mortality associated with low-risk lifestyle factors, assumed to be constant across age groups, were estimated from CKB. We fitted Cox proportional hazards regression models, with adjustment for education, marital status, hip circumference, family histories of heart attack and stroke (adjusted for analyses of all-cause and cardiovascular mortality), family history of cancer (adjusted for analyses of all-cause and cancer mortality), and menopausal status (adjusted for women only). To account for the potential region- and age-disparities in baseline hazard, the Cox model was stratified jointly by 10 study areas and age at baseline in a 5-year interval.

Population-based all-cause and cause-specific mortality rates in 2015 were obtained for the Chinese population by sex and 5-year age groups from the GBD website (http://ghdx.healthdata.org/gbd-results-tool). We desired to estimate the survival curves for the population across the entire age range. However, the all-cause mortality rates for ages 95 years and over are unrealistically lower than that for the age group 90-94 years, indicating possible age exaggeration at older ages.[4] We, therefore, applied a Poisson regression model with linear and quadratic terms for age (using the midpoints of 5-year age groups) to smooth and extrapolate the mortality rates beyond the upper bound age of 94 years. The Poisson model fitted observed mortality rates well in all combinations of analyses ($R^2 > 0.99$) (Figure S2). The sex- and age-specific population mortality rates were assumed to be constant in each 5-year interval.[5,6]

Population-based prevalence of low-risk lifestyle factors by sex and 5-year age intervals came from CNHS (2015).[7] National population census data from 2010 were used to calculate the sampling weights, and each participant was assigned a weight based on the study design. PROC SURVEYFREQ procedures in SAS were used to calculate the prevalences and their 95% confidence intervals (95% CIs). The sex- and age-specific prevalences of low-risk lifestyle factors were assumed to be constant in each 5-year interval until 84 years. For the population of 85 years onward, the prevalences were assumed to be the same as that in the 80-84 year age group due to insufficient numbers of participants aged ≥85 years in CNHS.

We used an algebraic transformation to infer the age-specific mortality rates appropriate for the reference group IR_{a1} by sex as:[8]

$$IR_{a1} = \frac{\mathrm{IR_a}}{\left(\mathrm{P_{a1}} + \sum_{\mathrm{j=2}}^{5} \mathrm{P_{aj}} \times \mathrm{HR_{aj}}\right)}$$

Where IR_a is the population mortality rate for age group *a*; P_{a1} is the age-specific prevalence of the reference group; P_{aj} is the age-specific prevalence of a lifestyle factor category *j*; and HR_{aj} is the HR for the corresponding lifestyle factor category *j* compared to the reference group (*j*=1). The age-specific mortality rates for each non-reference group were then inferred by multiplying the age-specific mortality rate for the reference group IR_{a1} and the HR_{aj}.

Finally, based on the sex- and age-specific mortality rate, life tables were built for each lifestyle factor category. Survival probability was set as 1 at the age of 30. The probability of surviving between ages *x* and *x*+1 was then estimated based on the probability of dying (mortality rate) between ages *x* and *x*+1, assuming that the survivor function declines linearly between ages *x* and *x*+1.[9,10] For the last open-ended age interval, the probability of surviving was set to 0. The life expectancy at any given age was derived by dividing the total person-years that would be lived beyond age *x* by the number of persons who survived to that age interval.[9] The gain in life expectancy according to different lifestyle factor categories was calculated as the difference in the life expectancy at any given age between the reference group and each non-reference category in turn (Figures 2 and 3B in manuscript).

The CI for life expectancy was estimated using @RISK 8.1 (Palisade Corp, Ithaca, NY), with 10,000 runs of Monte Carlo simulation (parametric bootstrapping), assuming that RR values obeyed the lognormal distribution, while the prevalence of lifestyle factors and the mortality rate data were normally distributed based on the central limit theorem.[11,12]

Statistical method used for estimating contributions of specific causes of death to the absolute difference in life expectancy

We applied Arriaga's decomposition method to estimate the contributions of specific causes of death to the absolute difference in life expectancy between participants adopting all five and 0-1 low-risk lifestyle factors. The analytical process consists of two steps:[13]

(1) Decomposition by each single-age group:

$$TE_x = \left[\frac{l_x^1}{l_0} \times \left(\frac{L_x^2}{l_x^2} - \frac{L_x^1}{l_x^1}\right)\right] + \left[\frac{T_{x+1}^2}{l_0} \times \left(\frac{l_x^1}{l_x^2} - \frac{l_{x+1}^1}{l_{x+1}^2}\right)\right]$$

For the last open-ended age interval, the contribution can be expressed as:

$$TE_x = \left[\frac{l_x^1}{l_0} \times \left(\frac{T_x^2}{l_x^2} - \frac{T_x^1}{l_x^1}\right)\right]$$

TE_x is the total contribution of age group x-x+1, l_x is the number of individuals alive at age x, l_0 is the hypothetical cohort size, L_x is the number of person-years lived within the single-age interval, T_x and T_{x+1} is the total number of person-years lived above age x and x+1, and l_{x+1} is the number of individuals alive at age x+1. "1" represents the reference group, that is participants adopting 0-1 low-risk lifestyle factors, and "2" represents those adopting all five low-risk lifestyle factors.

(2) Decomposition by cause of death within each single-age group:

$$TE_x^i = TE_x \times \left[\frac{m_{i,X}^2 - m_{i,X}^1}{m_X^2 - m_X^1}\right]$$

TE_x^i is the total contribution of age group x-x+1 due to cause i, TE_x is the total contribution of age group x-x+1, m_x is the all-cause mortality rate of age group x-x+1, $m_{i,x}$ is the specific mortality rate of age group x-x+1 due to cause i. "1" represents the reference group, that is participants adopting 0-1 low-risk lifestyle factors, and "2" represents those adopting all five low-risk lifestyle factors.

References

1. Du H, Bennett D, Li L, et al. Physical activity and sedentary leisure time and their associations with BMI, waist circumference, and percentage body fat in 0.5 million adults: the China Kadoorie Biobank study. *Am J Clin Nutr* 2013; **97**(3): 487-96.

2. Zhu N, Yu C, Guo Y, et al. Adherence to a healthy lifestyle and all-cause and cause-specific mortality in Chinese adults: a 10-year prospective study of 0.5 million people. *The international journal of behavioral nutrition and physical activity* 2019; **16**(1): 98.

3. Committee IR. Guidelines for data processing and analysis of the International Physical Activity Questionnaire (IPAQ)-short and long forms. *http://www ipaq ki se/scoring pdf* 2005.

4. Anderson RN. Method for constructing complete annual US life tables: National Ctr for Health Statistics; 1999.

5. Schoen R. Modeling multigroup populations: Springer Science & Business Media; 2013.

6. Pan XF, Li Y, Franco OH, Yuan JM, Pan A, Koh WP. Impact of Combined Lifestyle Factors on All-Cause and Cause-Specific Mortality and Life Expectancy in Chinese: The Singapore Chinese Health Study. *J Gerontol A Biol Sci Med Sci* 2020; **75**(11): 2193-9.

7. Brase CH, Brase CP. Understandable statistics: Concepts and methods, enhanced: Cengage Learning; 2016.

8. Woloshin S, Schwartz LM, Welch HG. The risk of death by age, sex, and smoking status in the United States: putting health risks in context. *Journal of the National Cancer Institute* 2008; **100**(12): 845-53.

9. Chiang CL, Organization WH. Life table and mortality analysis. 1979.

10. Arias E. United States life tables, 2008. *Natl Vital Stat Rep* 2012; **61**(3): 1-63.

11. Efron B, Tibshirani R. An Introduction to the Bootstrap. New York: Chapman & Hall/CRC. 1993.

12. Gardiner JC. The asymptotic distribution of mortality rates in competing risks analyses. *Scandinavian Journal of Statistics* 1982: 31-6.

13. Arriaga EE. Measuring and explaining the change in life expectancies. *Demography* 1984; **21**(1): 83-96.

Table S1. Numbers of the CNHS participants used to estimate the prevalence of individual or combined lifestyle factors*

	Smoking	Alcohol intake	Physical activity	Diet score†	Body shape	Combined factors
Men	78,221	80,650	80,509	33,881	77,315	31,726
30-34	4,836 (6.2)	4,877 (6.1)	4,869 (6.1)	1,955 (5.8)	4,485 (5.8)	1,796 (5.7)
35-39	5,520 (7.1)	5,584 (6.9)	5,573 (6.9)	2,279 (6.7)	5,255 (6.8)	2,143 (6.8)
40-44	8,186 (10.5)	8,278 (10.3)	8,261 (10.3)	3,392 (10.0)	7,826 (10.1)	3,198 (10.1)
45-49	10,463 (13.4)	10,663 (13.2)	10,642 (13.2)	4,401 (13.0)	10,222 (13.2)	4,169 (13.1)
50-54	10,916 (14.0)	11,189 (13.9)	11,163 (13.9)	4,700 (13.9)	10,772 (13.9)	4,455 (14.0)
55-59	9,716 (12.4)	10,018 (12.4)	10,002 (12.4)	4,231 (12.5)	9,674 (12.5)	3,985 (12.6)
60-64	11,013 (14.1)	11,493 (14.3)	11,485 (14.3)	4,967 (14.7)	11,130 (14.4)	4,634 (14.6)
65-69	8,279 (10.6)	8,667 (10.8)	8,656 (10.8)	3,707 (10.9)	8,440 (10.9)	3,481 (11.0)
70-74	5,018 (6.4)	5,345 (6.6)	5,329 (6.6)	2,300 (6.8)	5,169 (6.7)	2,089 (6.6)
75-79	2,995 (3.8)	3,170 (3.9)	3,165 (3.9)	1,350 (4.0)	3,044 (3.9)	1,244 (3.9)
80-84	1,279 (1.6)	1,366 (1.7)	1,364 (1.7)	599 (1.8)	1,298 (1.7)	532 (1.7)
Women	90,184	90,477	90,352	37,129	87,708	36,072
30-34	6,127 (6.8)	6,129 (6.8)	6,123 (6.8)	2,428 (6.5)	5,823 (6.6)	2,310 (6.4)
35-39	6,544 (7.3)	6,551 (7.2)	6,545 (7.2)	2,677 (7.2)	6,277 (7.2)	2,583 (7.2)
40-44	9,686 (10.7)	9,700 (10.7)	9,679 (10.7)	3,889 (10.5)	9,394 (10.7)	3,777 (10.5)
45-49	13,059 (14.5)	13,083 (14.5)	13,063 (14.5)	5,318 (14.3)	12,726 (14.5)	5,182 (14.4)
50-54	13,285 (14.7)	13,325 (14.7)	13,306 (14.7)	5,404 (14.6)	13,000 (14.8)	5,281 (14.6)
55-59	11,183 (12.4)	11,234 (12.4)	11,222 (12.4)	4,632 (12.5)	10,965 (12.5)	4,522 (12.5)
60-64	12,543 (13.9)	12,601 (13.9)	12,587 (13.9)	5,372 (14.5)	12,280 (14.0)	5,255 (14.6)
65-69	8,636 (9.6)	8,682 (9.6)	8,666 (9.6)	3,616 (9.7)	8,464 (9.7)	3,520 (9.8)
70-74	4,971 (5.5)	4,992 (5.5)	4,987 (5.5)	2,102 (5.7)	4,835 (5.5)	2,049 (5.7)
75-79	2,803 (3.1)	2,828 (3.1)	2,825 (3.1)	1,125 (3.0)	2,697 (3.1)	1,062 (2.9)
80-84	1,347 (1.5)	1,352 (1.5)	1,349 (1.5)	566 (1.5)	1,247 (1.4)	531 (1.5)
Total	168,405	171,127	170,861	71,010	165,023	67,798

*The numbers in parentheses indicate the percentage of participants in each age group for men and women, respectively.

†In the CNHS, 45 households in each village/neighborhood community were randomly selected. All the household members above the age of 18 participated in the survey, with information on smoking, alcohol intake, physical activity, weight, height, and waist circumference collected. Of the 45 households, 20 were randomly selected to fill out the food frequency questionnaire.

Table S2. Baseline characteristics of the CKB study participants according to number of low-risk lifestyle factors

	Number of low-risk lifestyle factors†					
	0	1	2	3	4	5
Men (n =199,238)						
No. of participants, n (%)*	5,175 (2.6)	30,908 (15.5)	70,862 (35.6)	68,905 (34.6)	21,992 (11.0)	1,396 (0.7)
Age, year (SD)	53.6 (10.5)	52.7 (10.6)	52.5 (10.7)	52.0 (10.9)	51.9 (11.1)	52.7 (11.3)
Urban area, n (%)	2,813 (54.4)	14,602 (47.2)	30,152 (42.6)	26,997 (39.2)	9,389 (42.7)	969 (69.4)
Middle school and above, n (%)	3,235 (57.6)	18,357 (57.2)	40,255 (57.0)	38,807 (57.6)	13,440 (61.1)	1,018 (68.6)
Married, n (%)	4,841 (93.7)	28,713 (93.0)	65,862 (93.0)	63,989 (92.8)	20,527 (93.1)	1,335 (95.2)
Having low-risk lifestyle factors, n (%)						
Non-smoking	-	1,689 (5.0)	12,846 (17.7)	28,093 (42.6)	20,008 (91.6)	-
Non-excessive alcohol intake	-	12,730 (44.1)	50,579 (72.1)	64,308 (92.9)	21,727 (98.7)	-
Being physically active	-	3,774 (13.4)	26,390 (37.9)	47,723 (68.3)	20,308 (91.9)	-
Healthy dietary habits	-	427 (1.2)	2,336 (3.3)	4,894 (7.5)	4,569 (20.2)	-
Ideal body shape	-	12,288 (39.1)	49,573 (69.5)	61,697 (89.6)	21,356 (97.2)	-
Prevalent hypertension, n (%)	2,750 (52.0)	13,224 (43.2)	25,519 (36.1)	22,511 (32.6)	7,104 (31.9)	461 (30.1)
Prevalent diabetes, n (%)	569 (9.3)	2,223 (6.8)	3,659 (5.2)	2,758 (4.2)	874 (4.0)	56 (3.1)
Family history of, n (%)						
Heart attack	213 (3.6)	1,128 (3.5)	2,130 (3.0)	2,086 (3.1)	658 (3.0)	43 (2.8)
Stroke	1,045 (19.2)	5,863 (18.7)	12,639 (17.9)	11,834 (17.3)	3,839 (17.5)	229 (16.7)
Cancer	1,034 (18.8)	5,585 (17.8)	12,060 (16.9)	11,191 (16.5)	3,566 (16.3)	233 (16.0)
Women (n = 287,971)						
No. of participants, n (%)*	135 (0.1)	2,206 (0.8)	46,819 (16.3)	126,894 (44.1)	103,546 (36.0)	8,371 (2.9)
Age, year (SD)	61.2 (10.1)	59.7 (10.2)	54.6 (10.4)	51.0 (10.3)	49.4 (9.9)	48.9 (9.7)
Urban area, n (%)	25 (18.5)	875 (39.7)	22,144 (47.3)	55,606 (43.8)	40,732 (39.3)	5,843 (69.8)
Middle school and above, n (%)	19 (28.4)	577 (33.2)	18,108 (39.2)	57,996 (43.4)	42,296 (44.3)	5,472 (55.6)
Married, n (%)	85 (80.1)	1,619 (86.0)	39,972 (88.9)	113,152 (89.2)	95,034 (90.2)	7,679 (90.8)
Having low-risk lifestyle factors, n (%)						
Non-smoking	-	499 (52.4)	43,287 (93.1)	124,534 (98.0)	103,464 (99.9)	-
Non-excessive alcohol intake	-	1,232 (74.6)	44,904 (95.6)	125,583 (98.9)	103,453 (99.9)	-
Being physically active	-	230 (7.7)	2,165 (5.5)	38,047 (32.2)	94,361 (89.7)	-
Healthy dietary habits	-	3 (0.1)	159 (0.3)	4,532 (3.5)	12,547 (13.7)	-
Ideal body shape	-	242 (10.8)	3,123 (6.4)	87,986 (67.5)	100,359 (97.3)	-
Prevalent hypertension, n (%)	57 (33.3)	972 (35.2)	21,174 (40.1)	40,455 (32.5)	27,956 (28.3)	2,132 (27.9)
Prevalent diabetes, n (%)	11 (5.9)	265 (8.0)	4,844 (8.5)	6,917 (5.5)	3,670 (4.0)	316 (3.7)
Family history of, n (%)						
Heart attack	5 (5.2)	52 (2.4)	1,526 (3.1)	4,088 (3.2)	3,100 (3.2)	369 (3.8)
Stroke	27 (21.6)	403 (16.8)	9,201 (17.8)	22,881 (17.6)	16,463 (17.3)	1,548 (18.1)
Cancer	22 (17.6)	412 (18.1)	8,394 (16.4)	20,932 (16.4)	15,991 (16.4)	1,517 (16.7)

Data were presented as mean (standard deviation) or number (percentage). Baseline characteristics were adjusted for age at enrollment and study areas, except in the cases where age or study area was the independent variable.

*The numbers in parentheses indicate the proportion of participants who had a different number of low-risk lifestyle factors.

†Low-risk lifestyle factors were defined as: never smoking or having stopped for reasons other than illness; less than daily drinking or drinking <30 g (men)/15 g (women) of pure alcohol per day (former drinkers excluded); engaging in an age- (<50 years, 50-59 years, and ≥60 years) and sex-specific median or higher level of physical activity; having at least 4 of the following dietary habits: eating fresh vegetables daily, eating fresh fruits daily, eating red meat 1-6 days per week, eating legumes ≥4 days

per week, eating fish ≥1 day per week; having a BMI between 18.5 and 27.9 kg/m^2 and a waist circumference <90 cm (men)/85 cm (women).

Table S3. Multivariable-adjusted hazard ratios (95% CIs) for all-cause and cause-specific mortality among men in CKB study

	HR (95% CI)			
	All causes	Cardiovascular diseases	Cancer	Chronic respiratory diseases[¶]
Smoking[*]				
Never	1.00 (Referent)	1.00 (Referent)	1.00 (Referent)	1.00 (Referent)
Former	1.07 (1.01-1.13)	0.98 (0.89-1.07)	1.16 (1.06-1.28)	1.16 (0.84-1.61)
Current (cigarettes or equivalents/day)				
1-9	1.29 (1.24-1.35)	1.28 (1.20-1.37)	1.36 (1.26-1.47)	1.72 (1.37-2.15)
10-19	1.24 (1.19-1.29)	1.19 (1.11-1.27)	1.40 (1.30-1.51)	1.38 (1.10-1.73)
≥20	1.35 (1.30-1.40)	1.23 (1.16-1.31)	1.62 (1.52-1.73)	1.47 (1.19-1.82)
Alcohol intake[†]				
Less than daily	1.07 (0.95-1.21)	1.10 (0.90-1.34)	0.99 (0.81-1.21)	0.88 (0.48-1.60)
Former	1.43 (1.26-1.62)	1.37 (1.11-1.68)	1.27 (1.03-1.57)	1.12 (0.60-2.09)
Current daily (g of pure alcohol/day)				
<15	1.00 (Referent)	1.00 (Referent)	1.00 (Referent)	1.00 (Referent)
15-29	1.09 (0.95-1.24)	1.06 (0.84-1.33)	1.14 (0.91-1.42)	0.77 (0.38-1.56)
30-59	1.16 (1.02-1.32)	1.11 (0.90-1.38)	1.17 (0.94-1.45)	0.79 (0.41-1.53)
≥60	1.44 (1.27-1.63)	1.36 (1.10-1.69)	1.55 (1.26-1.92)	1.04 (0.55-1.99)
Physical activity[‡]				
Quintile 1	1.00 (Referent)	1.00 (Referent)	1.00 (Referent)	1.00 (Referent)
Quintile 2	0.80 (0.77-0.84)	0.78 (0.74-0.83)	0.87 (0.81-0.93)	0.87 (0.70-1.09)
Quintile 3	0.77 (0.74-0.80)	0.71 (0.66-0.76)	0.89 (0.83-0.95)	0.75 (0.60-0.94)
Quintile 4	0.72 (0.69-0.75)	0.69 (0.64-0.74)	0.82 (0.76-0.88)	0.68 (0.54-0.84)
Quintile 5	0.65 (0.62-0.67)	0.60 (0.56-0.65)	0.78 (0.72-0.84)	0.53 (0.42-0.68)
Diet score[§]				
0-1	1.00 (Referent)	1.00 (Referent)	1.00 (Referent)	1.00 (Referent)
2	0.90 (0.87-0.93)	0.86 (0.81-0.91)	0.96 (0.91-1.02)	0.81 (0.68-0.97)
3	0.87 (0.83-0.90)	0.84 (0.78-0.90)	0.96 (0.89-1.03)	0.79 (0.63-0.99)
4	0.85 (0.79-0.91)	0.79 (0.71-0.89)	0.94 (0.85-1.05)	0.74 (0.49-1.12)
5	0.78 (0.65-0.94)	0.64 (0.46-0.89)	0.90 (0.68-1.20)	0.56 (0.14-2.27)
Body shape				
BMI <18.5 kg/m^2	1.39 (1.33-1.46)	1.10 (1.01-1.19)	1.34 (1.22-1.46)	2.30 (1.91-2.78)
BMI 18.5-27.9 kg/m^2, WC <90 cm (M)/85 cm (W)	1.00 (Referent)	1.00 (Referent)	1.00 (Referent)	1.00 (Referent)
BMI 18.5-27.9 kg/m^2, WC ≥90 cm (M)/85 cm (W)	1.19 (1.14-1.25)	1.29 (1.20-1.39)	1.05 (0.97-1.13)	1.49 (1.12-1.98)
BMI 28.0- kg/m^2, WC <90 cm (M)/85 cm (W)	1.33 (1.13-1.56)	1.65 (1.29-2.12)	1.05 (0.80-1.38)	1.79 (0.57-5.61)
BMI 28.0- kg/m^2, WC ≥90 cm (M)/85 cm (W)	1.32 (1.23-1.40)	1.58 (1.43-1.75)	0.92 (0.82-1.03)	1.75 (1.12-2.73)
No. of low-risk lifestyle factors[‖]				
0-1	1.00 (Referent)	1.00 (Referent)	1.00 (Referent)	1.00 (Referent)
2	0.74 (0.72-0.77)	0.80 (0.75-0.84)	0.75 (0.71-0.79)	0.68 (0.57-0.81)
3	0.59 (0.57-0.61)	0.62 (0.58-0.66)	0.63 (0.59-0.67)	0.43 (0.35-0.52)

	HR (95% CI)			
	All causes	Cardiovascular diseases	Cancer	Chronic respiratory diseases[¶]
4	0.49 (0.46-0.51)	0.53 (0.49-0.58)	0.48 (0.44-0.53)	0.31 (0.23-0.43)
5	0.39 (0.32-0.48)	0.45 (0.31-0.64)	0.36 (0.25-0.52)	0.50 (0.16-1.58)

BMI indicates body mass index; WC, waist circumference; HR, hazard ratio; CI, confidence interval; M, men; W, women.

Multivariable models were adjusted for education (no formal school, primary school, middle school, high school, college, or university or higher), marital status (married, widowed, divorced or separated, or never married), hip circumference (mm), family histories of heart attack and stroke (presence, absence, or unknown; adjusted for analyses of all-cause and cardiovascular mortality), and family history of cancer (presence, absence, or unknown; adjusted for analyses of all-cause and cancer mortality). In the analyses of individual lifestyle risk factors, all five lifestyle factors were included simultaneously in the same model.

[*]Former smokers referred to those having stopped smoking for reasons other than illness. Participants who had stopped smoking due to illness were classified as current smokers.

[†]Less than daily group included never-regular drinkers and current weekly drinkers. Former drinkers referred to those who used to drink at least once weekly but drank less than weekly at baseline.

[‡]Physical activity level was categorized based on age- (<50 years, 50-59 years, and ≥60 years) and sex-specific quintile of total physical activity level.

[§]Diet score was created based on the following criteria: eating fresh vegetables daily, eating fresh fruits daily, eating red meat 1-6 days per week, eating legumes ≥4 days per week, eating fish ≥1 day per week. For each food group, the participant who met the criterion received a score of 1, and otherwise, 0.

[||]The definition and classification of low-risk lifestyle factors were the same as in Table S2.

[¶]For the analysis of death from chronic respiratory diseases, participants with chronic obstructive pulmonary disease (COPD; n=17,136) or asthma (n=1,104) at baseline were further excluded, leaving 181,544 participants for the analysis.

Table S4. Multivariable-adjusted hazard ratios (95% CIs) for all-cause and cause-specific mortality among women in CKB study

	HR (95% CI)			
	All causes	Cardiovascular diseases	Cancer	Chronic respiratory diseases*
Smoking				
Never	1.00 (Referent)	1.00 (Referent)	1.00 (Referent)	1.00 (Referent)
Former	1.22 (1.06-1.40)	1.23 (0.99-1.53)	1.16 (0.88-1.53)	1.58 (0.83-3.02)
Current (cigarettes or equivalents/day)				
1-9	1.27 (1.18-1.37)	1.27 (1.13-1.44)	1.29 (1.12-1.50)	1.64 (1.19-2.27)
10-19	1.34 (1.21-1.49)	1.25 (1.06-1.49)	1.46 (1.21-1.76)	1.94 (1.27-2.98)
≥20	1.48 (1.29-1.70)	1.59 (1.29-1.95)	1.47 (1.13-1.92)	1.32 (0.64-2.71)
Alcohol intake				
Less than daily	1.22 (0.96-1.56)	1.30 (0.87-1.94)	0.91 (0.62-1.34)	0.82 (0.26-2.56)
Former	1.43 (1.09-1.86)	1.47 (0.95-2.29)	0.91 (0.58-1.43)	0.98 (0.28-3.43)
Current daily (g of pure alcohol/day)				
<15	1.00 (Referent)	1.00 (Referent)	1.00 (Referent)	1.00 (Referent)
15-29	1.05 (0.77-1.45)	1.47 (0.89-2.41)	0.76 (0.44-1.32)	0.95 (0.24-3.83)
30-59	1.14 (0.82-1.59)	1.38 (0.80-2.37)	0.88 (0.50-1.55)	1.15 (0.29-4.66)
≥60	0.95 (0.63-1.44)	0.82 (0.38-1.78)	0.86 (0.43-1.72)	1.19 (0.24-6.00)
Physical activity				
Quintile 1	1.00 (Referent)	1.00 (Referent)	1.00 (Referent)	1.00 (Referent)
Quintile 2	0.83 (0.79-0.86)	0.78 (0.73-0.83)	0.97 (0.90-1.05)	0.86 (0.68-1.08)
Quintile 3	0.75 (0.72-0.78)	0.69 (0.64-0.74)	0.93 (0.86-1.01)	0.59 (0.46-0.77)
Quintile 4	0.69 (0.66-0.73)	0.64 (0.59-0.69)	0.94 (0.86-1.02)	0.56 (0.43-0.73)
Quintile 5	0.63 (0.59-0.66)	0.60 (0.55-0.65)	0.84 (0.77-0.92)	0.55 (0.42-0.72)
Diet score				
0-1	1.00 (Referent)	1.00 (Referent)	1.00 (Referent)	1.00 (Referent)
2	0.90 (0.87-0.94)	0.91 (0.85-0.96)	0.92 (0.85-0.99)	0.76 (0.62-0.93)
3	0.81 (0.77-0.85)	0.79 (0.73-0.86)	0.87 (0.79-0.95)	0.72 (0.54-0.95)
4	0.75 (0.70-0.81)	0.70 (0.62-0.80)	0.88 (0.78-0.99)	0.62 (0.37-1.05)
5	0.71 (0.58-0.88)	0.64 (0.44-0.93)	0.67 (0.48-0.93)	0.41 (0.06-2.98)
Body shape				
BMI <18.5 kg/m^2	1.33 (1.26-1.40)	1.17 (1.07-1.28)	1.19 (1.06-1.34)	1.96 (1.54-2.50)
BMI 18.5-27.9 kg/m^2, WC <90 cm (M)/85 cm (W)	1.00 (Referent)	1.00 (Referent)	1.00 (Referent)	1.00 (Referent)
BMI 18.5-27.9 kg/m^2, WC ≥90 cm (M)/85 cm (W)	1.16 (1.11-1.21)	1.24 (1.16-1.32)	0.96 (0.89-1.04)	1.33 (1.05-1.70)
BMI 28.0- kg/m^2, WC <90 cm (M)/85 cm (W)	1.10 (0.94-1.29)	1.31 (1.02-1.68)	0.94 (0.72-1.23)	2.04 (0.75-5.52)
BMI 28.0- kg/m^2, WC ≥90 cm (M)/85 cm (W)	1.43 (1.34-1.52)	1.62 (1.48-1.78)	1.10 (0.99-1.23)	1.70 (1.16-2.49)
No. of low-risk lifestyle factors				
0-1	1.00 (Referent)	1.00 (Referent)	1.00 (Referent)	1.00 (Referent)
2	0.82 (0.74-0.90)	0.78 (0.68-0.90)	0.90 (0.74-1.11)	0.54 (0.37-0.80)
3	0.63 (0.58-0.70)	0.59 (0.51-0.68)	0.81 (0.66-0.99)	0.41 (0.28-0.60)

	HR (95% CI)			
	All causes	Cardiovascular diseases	Cancer	Chronic respiratory diseases*
4	0.51 (0.46-0.56)	0.47 (0.40-0.54)	0.78 (0.63-0.95)	0.20 (0.14-0.30)
5	0.42 (0.36-0.50)	0.37 (0.28-0.49)	0.64 (0.49-0.85)	0.21 (0.07-0.61)

BMI indicates body mass index; WC, waist circumference; HR, hazard ratio; CI, confidence interval; M, men; W, women.

The classification of individual lifestyle factors and definition of low-risk lifestyle factors were the same as in Table S3. Multivariable models were adjusted for education, marital status, hip circumference, family histories of heart attack, stroke, and cancer, and menopausal status. In the analyses of individual lifestyle risk factors, all five lifestyle factors were included simultaneously in the same model.

*For the analysis of death from chronic respiratory diseases, participants with chronic obstructive pulmonary disease (COPD; n=17,407) or asthma (n=1,424) at baseline were further excluded, leaving 269,689 participants for the analysis.

Table S5. Sensitivity analyses for all-cause and cause-specific mortality by excluding deaths during the first two years of follow-up in CKB study

	All causes			Cardiovascular diseases			Cancer		
	Deaths	Deaths /PYs (/1,000)	HR (95% CI)	Deaths	Deaths /PYs (/1,000)	HR (95% CI)	Deaths	Deaths /PYs (/1,000)	HR (95% CI)
Smoking									
Never	19,718	5.4	1.00 (Referent)	8,165	2.3	1.00 (Referent)	6,162	1.7	1.00 (Referent)
Former	1,732	11.5	1.12 (1.06-1.18)	639	4.3	1.03 (0.95-1.13)	582	3.9	1.16 (1.06-1.28)
Current (cigarettes or equivalents/day)									
1-9	4,242	14.5	1.30 (1.26-1.36)	1,815	6.2	1.29 (1.22-1.37)	1,212	4.1	1.33 (1.24-1.43)
10-19	4,369	10.7	1.27 (1.22-1.32)	1,623	4.0	1.21 (1.14-1.29)	1,490	3.6	1.40 (1.30-1.50)
≥20	8,082	9.8	1.43 (1.38-1.48)	2,617	3.2	1.32 (1.24-1.40)	3,126	3.8	1.64 (1.55-1.75)
Alcohol intake									
Less than daily	29,949	6.5	1.06 (0.95-1.19)	12,178	2.6	1.08 (0.90-1.29)	9,447	2.0	0.94 (0.78-1.13)
Former	2,960	15.7	1.35 (1.20-1.52)	1,069	5.6	1.31 (1.08-1.59)	901	4.8	1.14 (0.93-1.38)
Current daily (g of pure alcohol/day)									
<15	315	12.5	1.00 (Referent)	119	4.7	1.00 (Referent)	112	4.4	1.00 (Referent)
15-29	994	10.9	1.12 (0.98-1.27)	347	3.8	1.10 (0.90-1.36)	392	4.3	1.11 (0.90-1.37)
30-59	1,646	10.5	1.19 (1.05-1.34)	518	3.3	1.15 (0.94-1.40)	678	4.3	1.15 (0.94-1.41)
≥60	2,279	11.1	1.40 (1.24-1.58)	628	3.1	1.33 (1.09-1.62)	1,042	5.1	1.48 (1.21-1.80)
Physical activity									
Quintile 1	9,616	9.2	1.00 (Referent)	4,101	3.9	1.00 (Referent)	2,797	2.7	1.00 (Referent)
Quintile 2	7,872	7.5	0.84 (0.81-0.86)	3,234	3.1	0.81 (0.77-0.85)	2,490	2.4	0.93 (0.88-0.98)
Quintile 3	7,323	6.9	0.79 (0.76-0.81)	2,782	2.6	0.72 (0.69-0.76)	2,473	2.3	0.93 (0.88-0.99)
Quintile 4	7,031	6.6	0.74 (0.72-0.77)	2,571	2.4	0.69 (0.66-0.73)	2,467	2.3	0.92 (0.87-0.97)
Quintile 5	6,301	5.9	0.69 (0.67-0.71)	2,171	2.0	0.63 (0.60-0.67)	2,345	2.2	0.87 (0.82-0.92)
Diet score									
0-1	10,563	9.3	1.00 (Referent)	4,789	4.2	1.00 (Referent)	2,918	2.6	1.00 (Referent)
2	16,005	7.4	0.91 (0.88-0.93)	5,966	2.8	0.89 (0.85-0.93)	5,278	2.4	0.95 (0.91-1.00)
3	9,397	6.0	0.85 (0.82-0.87)	3,374	2.1	0.83 (0.78-0.87)	3,473	2.2	0.93 (0.87-0.98)
4	1,974	5.1	0.80 (0.76-0.84)	666	1.7	0.75 (0.68-0.82)	821	2.1	0.92 (0.84-1.00)

	All causes			Cardiovascular diseases			Cancer		
	Deaths	Deaths /PYs (/1,000)	HR (95% CI)	Deaths	Deaths /PYs (/1,000)	HR (95% CI)	Deaths	Deaths /PYs (/1,000)	HR (95% CI)
5	204	5.0	0.75 (0.66-0.87)	64	1.6	0.65 (0.51-0.83)	82	2.0	0.82 (0.65-1.02)
Body shape									
BMI <18.5 kg/m^2	3,653	16.7	1.32 (1.27-1.38)	1,280	5.8	1.11 (1.04-1.18)	863	3.9	1.21 (1.12-1.31)
BMI 18.5-27.9 kg/m^2, WC <90 cm (M)/85 cm (W)	24,936	6.6	1.00 (Referent)	9,277	2.4	1.00 (Referent)	8,717	2.3	1.00 (Referent)
BMI 18.5-27.9 kg/m^2, WC ≥90 cm (M)/85 cm (W)	5,943	8.0	1.19 (1.15-1.23)	2,662	3.6	1.31 (1.25-1.38)	1,828	2.5	0.97 (0.92-1.03)
BMI 28.0- kg/m^2, WC <90 cm (M)/85 cm (W)	287	4.8	1.22 (1.09-1.38)	118	2.0	1.45 (1.21-1.74)	104	1.7	1.01 (0.83-1.23)
BMI 28.0- kg/m^2, WC ≥90 cm (M)/85 cm (W)	3,324	6.9	1.39 (1.32-1.45)	1,522	3.1	1.64 (1.53-1.76)	1,060	2.2	1.00 (0.92-1.08)
No. of low-risk lifestyle factors									
0-1	5,576	13.9	1.00 (Referent)	2,048	5.1	1.00 (Referent)	1,852	4.6	1.00 (Referent)
2	12,367	9.9	0.75 (0.73-0.78)	5,064	4.0	0.78 (0.74-0.82)	3,858	3.1	0.76 (0.72-0.80)
3	13,927	6.5	0.59 (0.58-0.62)	5,492	2.6	0.60 (0.56-0.63)	4,517	2.1	0.65 (0.61-0.69)
4	5,979	4.3	0.50 (0.48-0.52)	2,167	1.6	0.50 (0.47-0.53)	2,219	1.6	0.60 (0.56-0.64)
5	294	2.7	0.39 (0.35-0.44)	88	0.8	0.37 (0.30-0.46)	126	1.2	0.48 (0.40-0.58)

BMI indicates body mass index; WC, waist circumference; PYs, person-years; HR, hazard ratio; CI, confidence interval; M, men; W, women.

The classification of individual lifestyle factors and definition of low-risk lifestyle factors were the same as in Table S3. Multivariable models were adjusted for sex, education, marital status, hip circumference, family histories of heart attack, stroke, and cancer. In the analyses of individual lifestyle risk factors, all five lifestyle factors were included simultaneously in the same model.

Table S6. Sensitivity analyses for mortality of chronic respiratory diseases by excluding deaths during the first two years of follow-up in CKB study*

	Deaths	Deaths /PYs (/1,000)	HR (95% CI)
Smoking			
Never	596	0.2	1.00 (Referent)
Former	54	0.4	1.31 (0.97-1.77)
Current (cigarettes or equivalents/day)			
1-9	220	0.8	1.83 (1.52-2.21)
10-19	183	0.5	1.52 (1.24-1.87)
≥20	244	0.3	1.61 (1.32-1.97)
Alcohol intake			
Less than daily	1,030	0.2	1.01 (0.56-1.84)
Former	107	0.7	1.26 (0.68-2.36)
Current daily (g of pure alcohol/day)			
<15	11	0.5	1.00 (Referent)
15-29	33	0.4	1.05 (0.53-2.08)
30-59	47	0.3	0.99 (0.51-1.91)
≥60	69	0.4	1.17 (0.61-2.23)
Physical activity			
Quintile 1	283	0.3	1.00 (Referent)
Quintile 2	272	0.3	0.88 (0.74-1.04)
Quintile 3	233	0.2	0.74 (0.62-0.88)
Quintile 4	255	0.3	0.65 (0.54-0.78)
Quintile 5	254	0.3	0.60 (0.50-0.73)
Diet score			
0-1	465	0.4	1.00 (Referent)
2	529	0.3	0.81 (0.70-0.93)
3	259	0.2	0.76 (0.63-0.91)
4	41	0.1	0.68 (0.48-0.95)
5	3	0.1	0.53 (0.17-1.66)
Body shape			
BMI <18.5 kg/m^2	277	1.5	2.00 (1.71-2.35)
BMI 18.5-27.9 kg/m^2, WC <90 cm (M)/85 cm (W)	775	0.2	1.00 (Referent)
BMI 18.5-27.9 kg/m^2, WC ≥90 cm (M)/85 cm (W)	167	0.2	1.46 (1.20-1.76)
BMI 28.0- kg/m^2, WC <90 cm (M)/85 cm (W)	6	0.1	1.79 (0.80-4.04)
BMI 28.0- kg/m^2, WC ≥90 cm (M)/85 cm (W)	72	0.2	2.01 (1.50-2.69)
No. of low-risk lifestyle factors			
0-1	207	0.6	1.00 (Referent)
2	442	0.4	0.66 (0.56-0.78)
3	499	0.3	0.47 (0.39-0.56)
4	143	0.1	0.27 (0.21-0.33)
5	6	0.1	0.29 (0.13-0.67)

BMI indicates body mass index; WC, waist circumference; PYs, person-years; HR, hazard ratio; CI, confidence interval; M, men; W, women.

The classification of individual lifestyle factors and definition of low-risk lifestyle factors were the same as in Table S3. Multivariable model was adjusted for sex, education, marital status, and hip circumference. In the analyses of individual lifestyle risk factors, all five lifestyle factors were included simultaneously in the same model.

*Participants with chronic obstructive pulmonary disease (COPD) or asthma at baseline were excluded from the analysis population in Table S5.

Table S7. Competing risk analyses for cause-specific mortality by using Fine and Gray regression model

	Sub-distribution HRs (95% CIs)		
	Cardiovascular diseases	Cancer	Chronic respiratory diseases*
Smoking			
Never	1.00 (Referent)	1.00 (Referent)	1.00 (Referent)
Former	1.02 (0.95-1.11)	1.15 (1.06-1.25)	1.29 (0.97-1.73)
Current (cigarettes or equivalents/day)			
1-9	1.20 (1.14-1.27)	1.30 (1.22-1.38)	1.71 (1.43-2.04)
10-19	1.15 (1.08-1.22)	1.35 (1.27-1.44)	1.48 (1.21-1.80)
≥20	1.22 (1.16-1.29)	1.54 (1.46-1.63)	1.54 (1.27-1.86)
Alcohol intake			
Less than daily	1.05 (0.89-1.25)	0.92 (0.77-1.09)	0.77 (0.46-1.30)
Former	1.24 (1.04-1.48)	1.13 (0.95-1.36)	0.94 (0.55-1.63)
Current daily (g of pure alcohol/day)			
<15	1.00 (Referent)	1.00 (Referent)	1.00 (Referent)
15-29	1.09 (0.90-1.32)	1.06 (0.87-1.28)	0.75 (0.41-1.39)
30-59	1.10 (0.91-1.32)	1.10 (0.91-1.32)	0.73 (0.41-1.32)
≥60	1.25 (1.04-1.51)	1.41 (1.18-1.69)	0.84 (0.48-1.48)
Physical activity			
Quintile 1	1.00 (Referent)	1.00 (Referent)	.1.00 (Referent)
Quintile 2	0.81 (0.78-0.85)	0.94 (0.89-0.98)	0.94 (0.80-1.09)
Quintile 3	0.73 (0.69-0.76)	0.93 (0.89-0.98)	0.74 (0.63-0.87)
Quintile 4	0.69 (0.66-0.72)	0.90 (0.85-0.95)	0.68 (0.58-0.80)
Quintile 5	0.62 (0.59-0.66)	0.83 (0.79-0.88)	0.59 (0.50-0.71)
Diet score			
0-1	1.00 (Referent)	1.00 (Referent)	1.00 (Referent)
2	0.90 (0.86-0.93)	0.96 (0.92-1.01)	0.80 (0.70-0.92)
3	0.82 (0.78-0.86)	0.94 (0.89-0.99)	0.76 (0.64-0.91)
4	0.74 (0.69-0.81)	0.93 (0.86-1.01)	0.68 (0.50-0.94)
5	0.66 (0.52-0.83)	0.83 (0.67-1.02)	0.54 (0.17-1.68)
Body shape			
BMI <18.5 kg/m^2	1.02 (0.96-1.08)	1.18 (1.10-1.26)	2.03 (1.76-2.35)
BMI 18.5-27.9 kg/m^2, WC <90 cm (M)/85 cm (W)	1.00 (Referent)	1.00 (Referent)	1.00 (Referent)
BMI 18.5-27.9 kg/m^2, WC ≥90 cm (M)/85 cm (W)	1.27 (1.22-1.33)	0.98 (0.93-1.03)	1.42 (1.19-1.70)
BMI 28.0- kg/m^2, WC <90 cm (M)/85 cm (W)	1.58 (1.34-1.87)	1.05 (0.87-1.27)	1.92 (0.91-4.06)
BMI 28.0- kg/m^2, WC ≥90 cm (M)/85 cm (W)	1.66 (1.56-1.77)	1.03 (0.95-1.11)	1.82 (1.40-2.37)
No. of low-risk lifestyle factors			
0-1	1.00 (Referent)	1.00 (Referent)	1.00 (Referent)
2	0.83 (0.79-0.87)	0.78 (0.74-0.82)	0.67 (0.57-0.79)
3	0.65 (0.62-0.68)	0.68 (0.64-0.71)	0.46 (0.40-0.55)

	Sub-distribution HRs (95% CIs)		
	Cardiovascular diseases	Cancer	Chronic respiratory diseases*
4	0.54 (0.51-0.58)	0.61 (0.57-0.65)	0.27 (0.22-0.34)
5	0.41 (0.34-0.51)	0.50 (0.42-0.59)	0.33 (0.16-0.70)

BMI indicates body mass index; WC, waist circumference; HR, hazard ratio; CI, confidence interval; M, men; W, women.
The classification of individual lifestyle factors and definition of low-risk lifestyle factors were the same as in Table S3. Multivariable model was adjusted for the same covariates as Table S5. In the analyses of individual lifestyle risk factors, all five lifestyle factors were included simultaneously in the same model.
*For the analysis of death from chronic respiratory diseases, participants with chronic obstructive pulmonary disease (COPD; n=34,543) or asthma (n=2,528) at baseline were further excluded.

Table S8. Years of life gained at age 30, 50, and 65 years by the number of low-risk lifestyle factors.

	Men	Women
Years of life gained (95% CIs), 30 y		
0-1	Referent	Referent
2	2.8 (2.5, 3.2)	1.9 (1.0, 2.8)
3	4.9 (4.6, 5.3)	4.2 (3.3, 5.2)
4	6.8 (6.3, 7.3)	6.3 (5.3, 7.4)
5	8.8 (6.8, 10.7)	8.1 (6.5, 9.9)
Years of life gained (95% CIs), 50 y		
0-1	Referent	Referent
2	2.4 (2.1, 2.7)	1.7 (0.9, 2.5)
3	4.3 (4.0, 4.6)	3.9 (3.1, 4.8)
4	5.9 (5.5, 6.4)	5.9 (4.9, 6.8)
5	7.7 (6.0, 9.5)	7.6 (6.0, 9.3)
Years of life gained (95% CIs), 65 y		
0-1	Referent	Referent
2	1.8 (1.6, 2.0)	1.4 (0.7, 2.1)
3	3.3 (3.0, 3.5)	3.3 (2.6, 4.1)
4	4.6 (4.2, 5.0)	5.0 (4.1, 5.9)
5	6.1 (4.6, 7.6)	6.6 (5.2, 8.1)

CI indicates confidence interval.
The definition of low-risk lifestyle factors was the same as in Table S2.

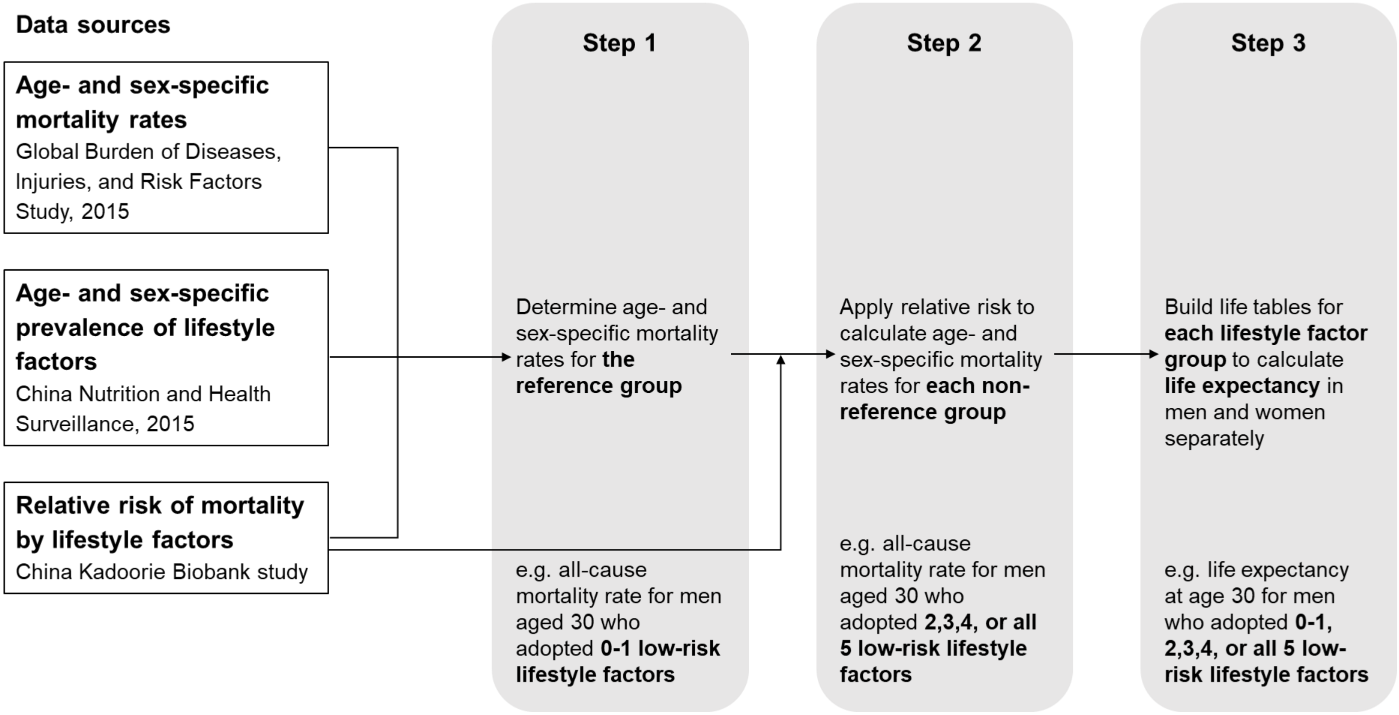

Figure S1. Data sources and steps used to calculate the life expectancy of each lifestyle factor group.

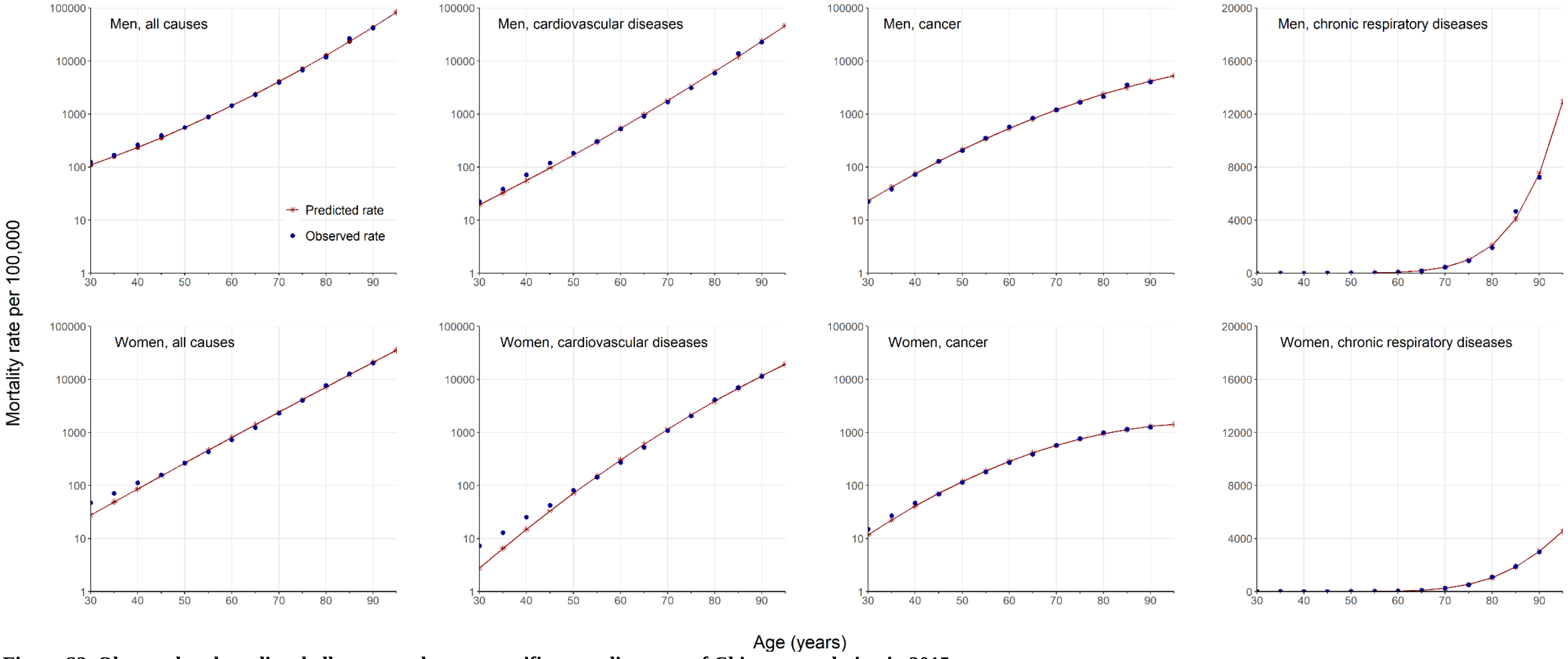

Figure S2. Observed and predicted all-cause and cause-specific mortality rates of Chinese population in 2015.
We used a Poisson regression model with linear and quadratic terms for the midpoints of 5-year age groups to smooth and extrapolate the mortality rates beyond the upper bound age of 94 years. The coefficients of determination (R^2) were over 0.99 for all combinations of analyses.

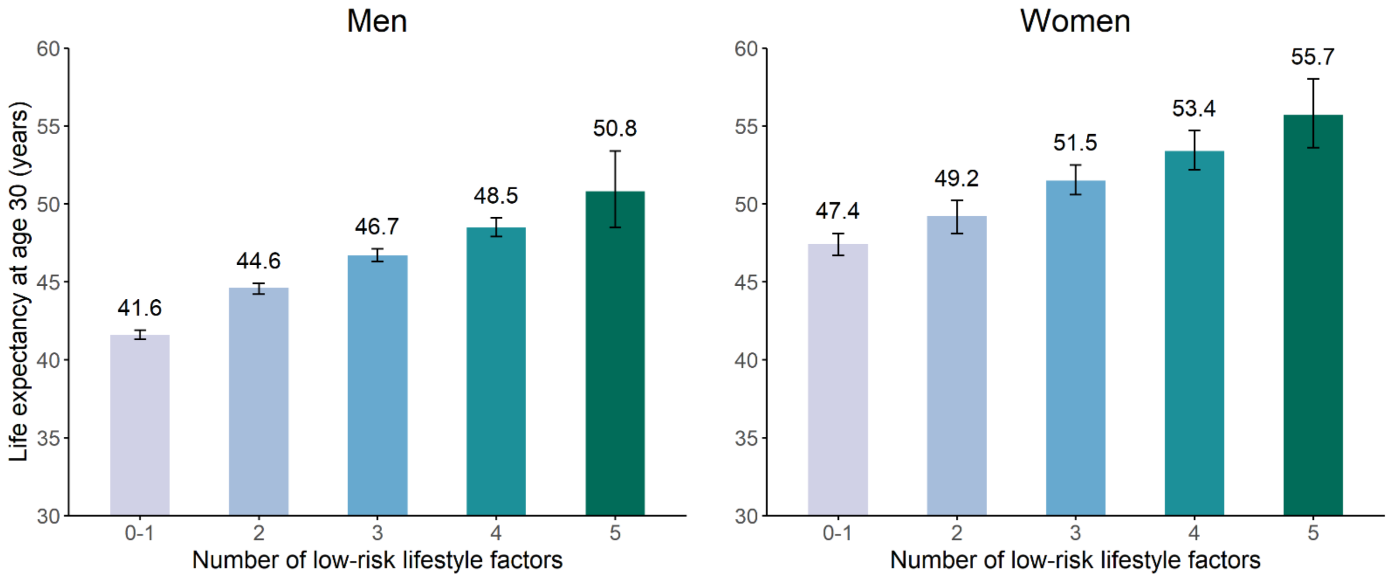

Figure S3. Sensitivity analyses of estimated life expectancy at age 30 according to the number of low-risk lifestyle factors by using sex- and age-at-risk specific hazard ratios.
The definition of low-risk lifestyle factors was the same as in Table S2.

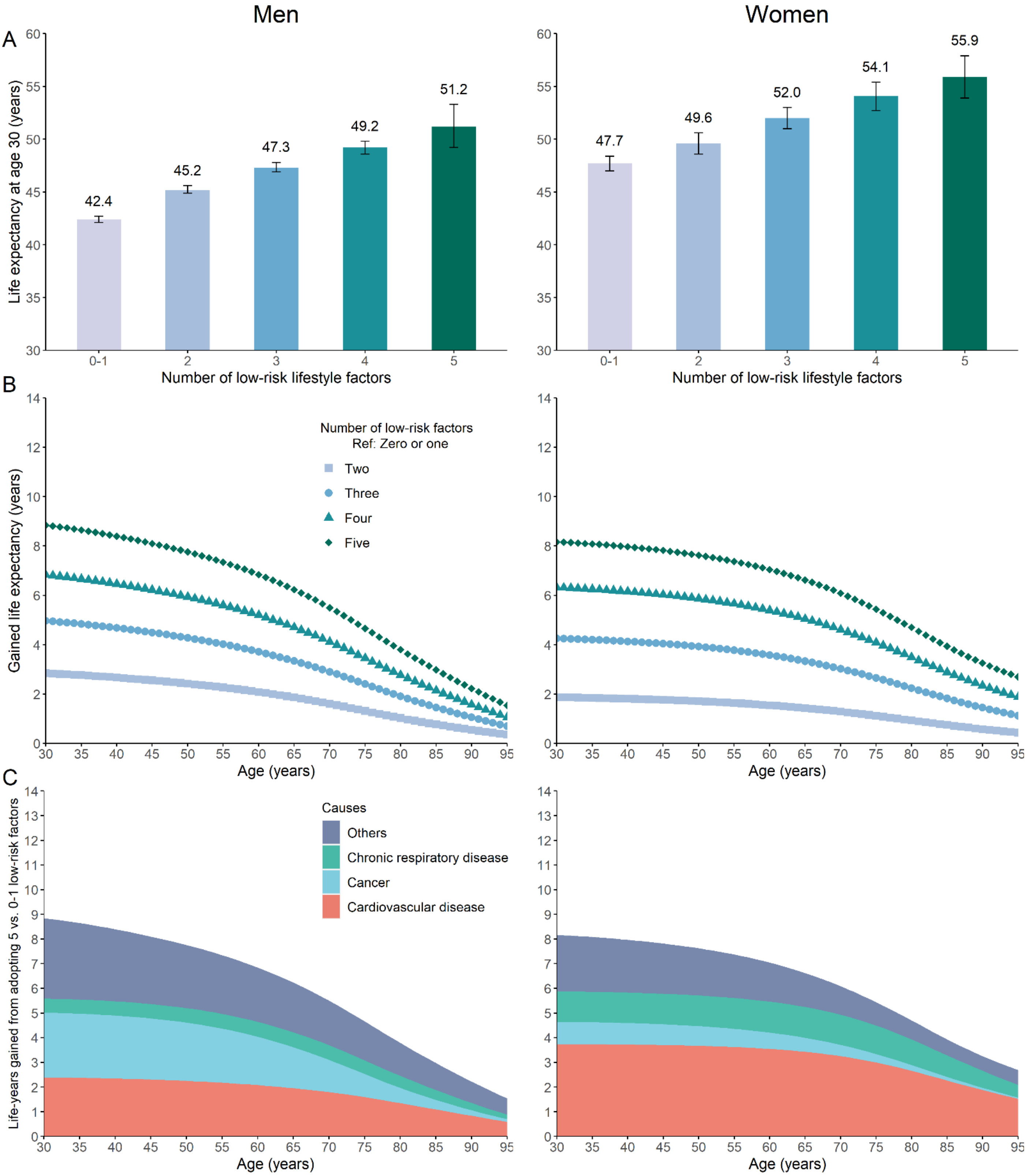

Figure S4. Sensitivity analyses of estimated life expectancy and years of life gained by the number of low-risk lifestyle factors and attribution of the causes of death using mortality data from 2019.

(A) Estimated life expectancy at age 30 by the number of low-risk lifestyle factors; (B) Gained age-specific life expectancy from adopting low-risk lifestyle; (C) Estimated years of life gained from adopting five versus zero or one low-risk lifestyle factor attributable to reduced death from cardiovascular disease, cancer, chronic respiratory disease, and other causes.

The definition of low-risk lifestyle factors was the same as in Table S2.

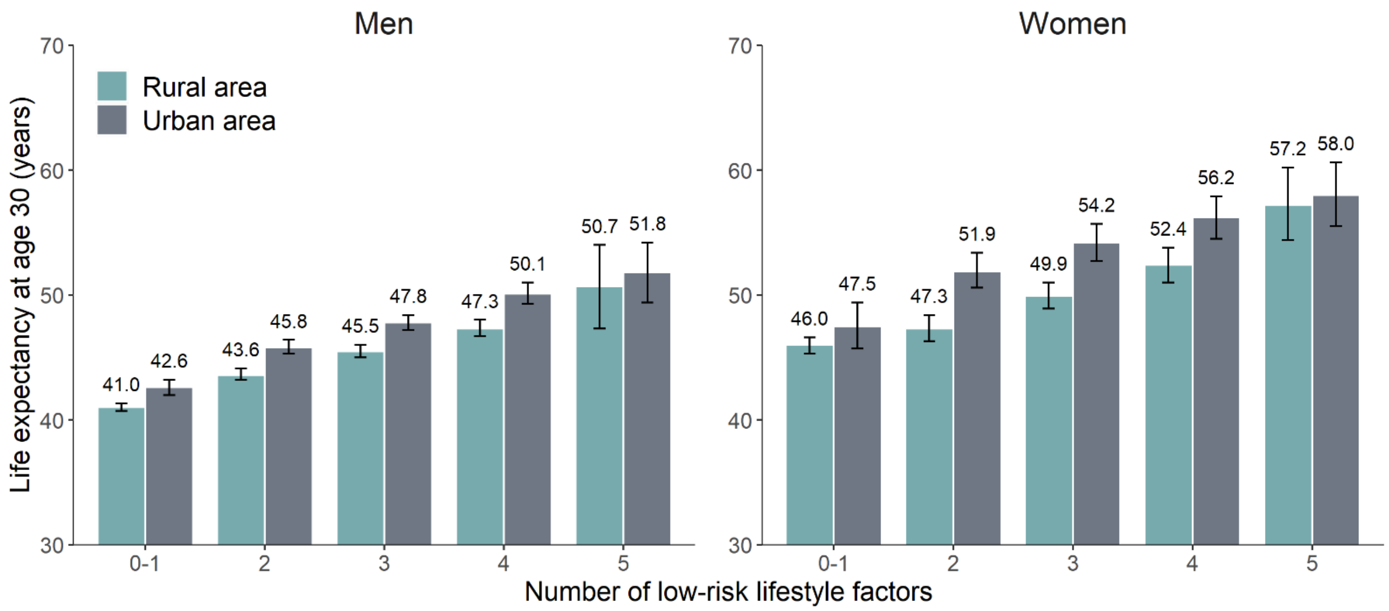

Figure S5. Estimated life expectancy at age 30 according to the number of low-risk factors stratified by residence. The definition of low-risk lifestyle factors was the same as in Table S2.

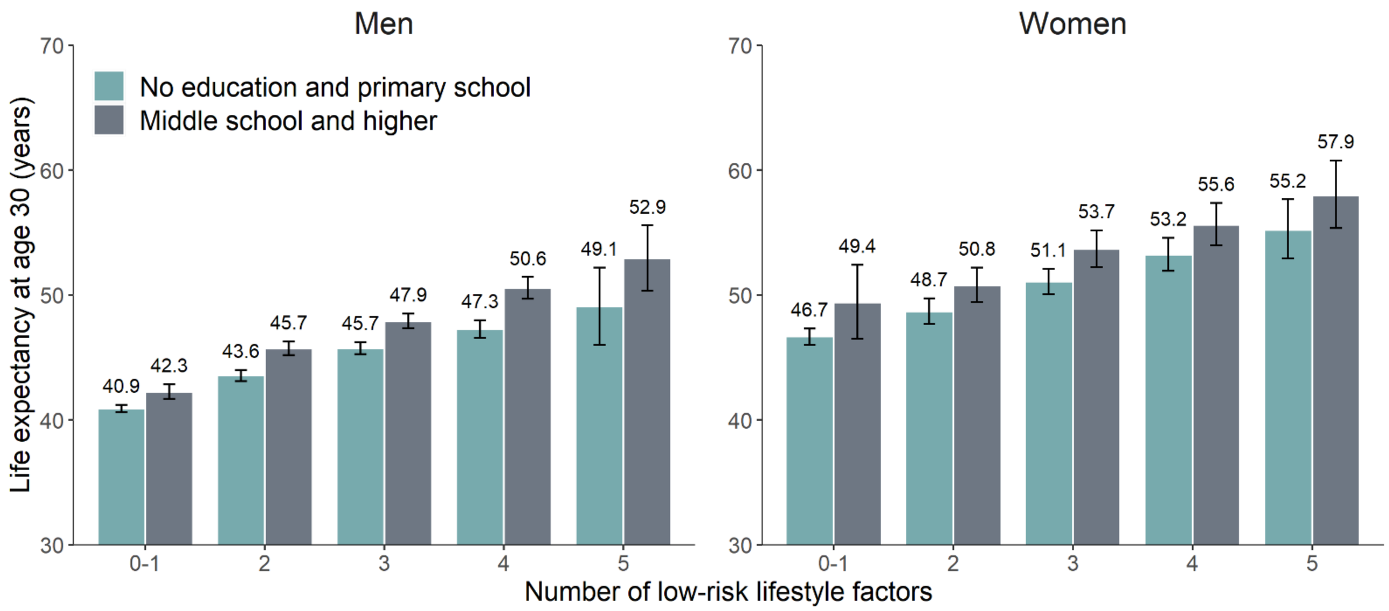

Figure S6. Estimated life expectancy at age 30 according to the number of low-risk factors stratified by education level. The definition of low-risk lifestyle factors was the same as in Table S2.

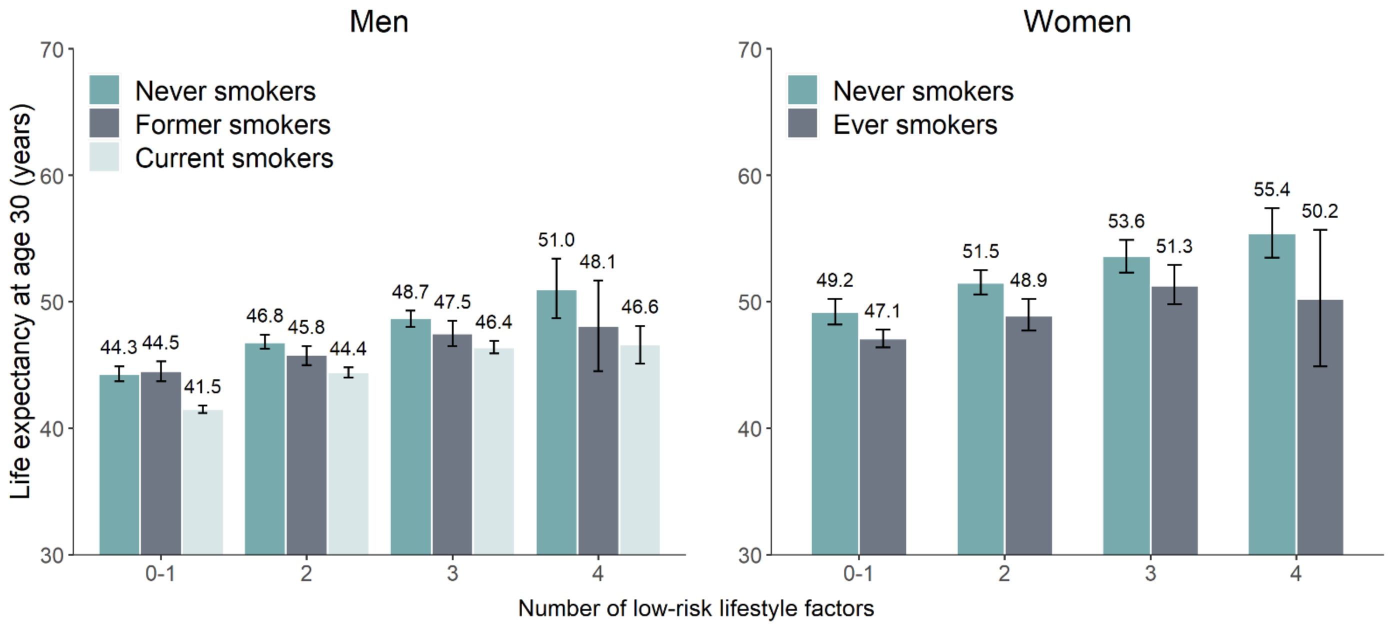

Figure S7. Estimated life expectancy at age 30 according to the number of low-risk factors stratified by smoking status. The definition of low-risk lifestyle factors was the same as in Table S2. Former smokers referred to those having stopped smoking for reasons other than illness. Participants who had stopped smoking due to illness were classified as current smokers. Due to low smoking prevalence among women, former and current smokers were combined into a group of ever smokers.

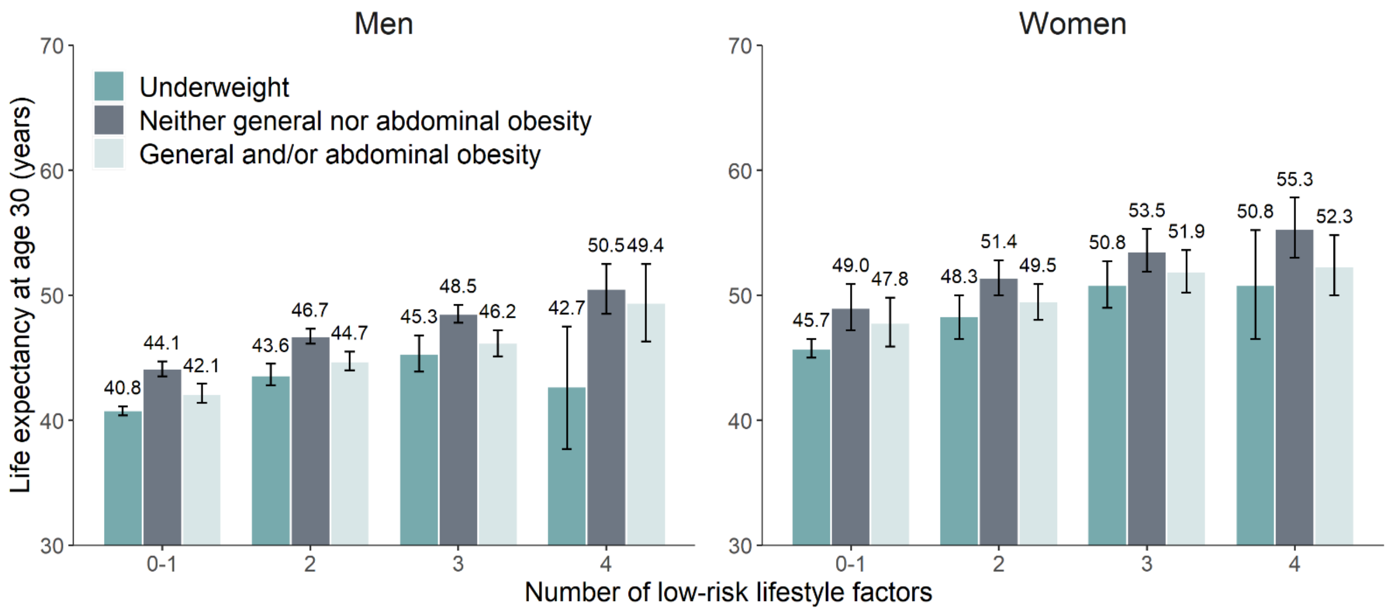

Figure S8. Estimated life expectancy at age 30 according to the number of low-risk factors stratified by obesity status. The definition of low-risk lifestyle factors was the same as in Table S2. Underweight: BMI <18.5 kg/m^2; neither general nor abdominal obesity: BMI 18.5-27.9 kg/m^2 and waist circumference <90 cm (men)/85 cm (women); general and/or abdominal obesity: BMI ≥28.0 kg/m^2 and/or waist circumference ≥90 cm (men)/85 cm (women).

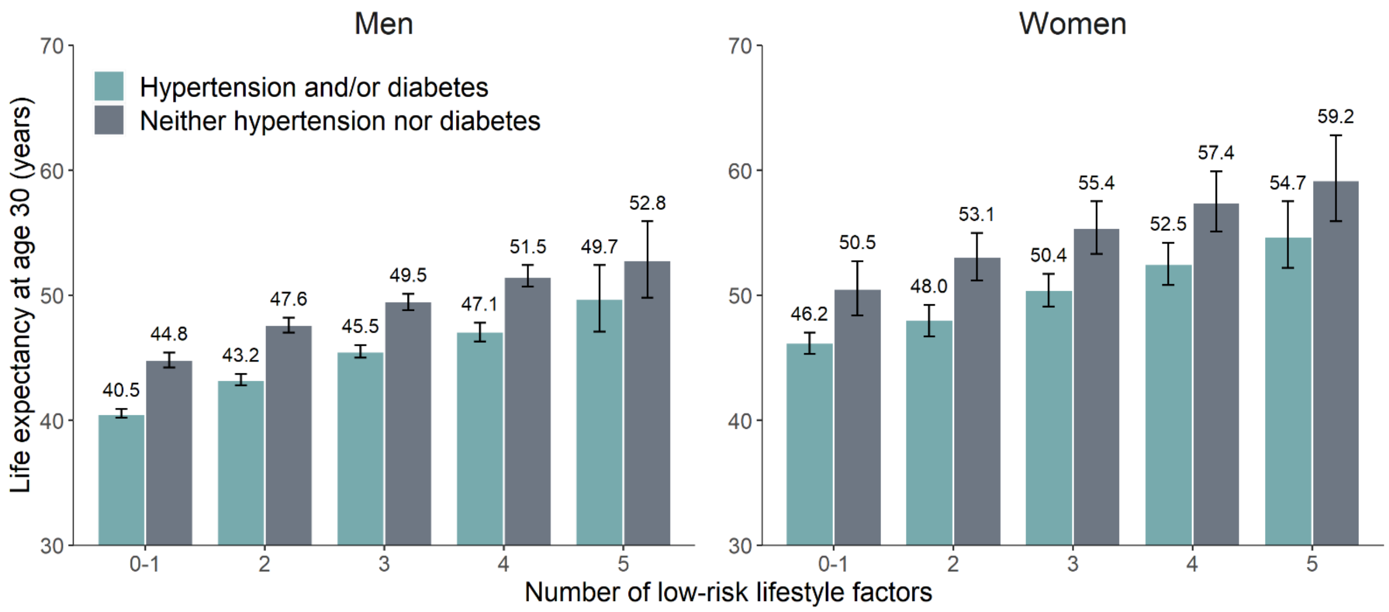

Figure S9. Estimated life expectancy at age 30 according to the number of low-risk factors stratified by baseline status of hypertension and diabetes.

The definition of low-risk lifestyle factors was the same as in Table S2.

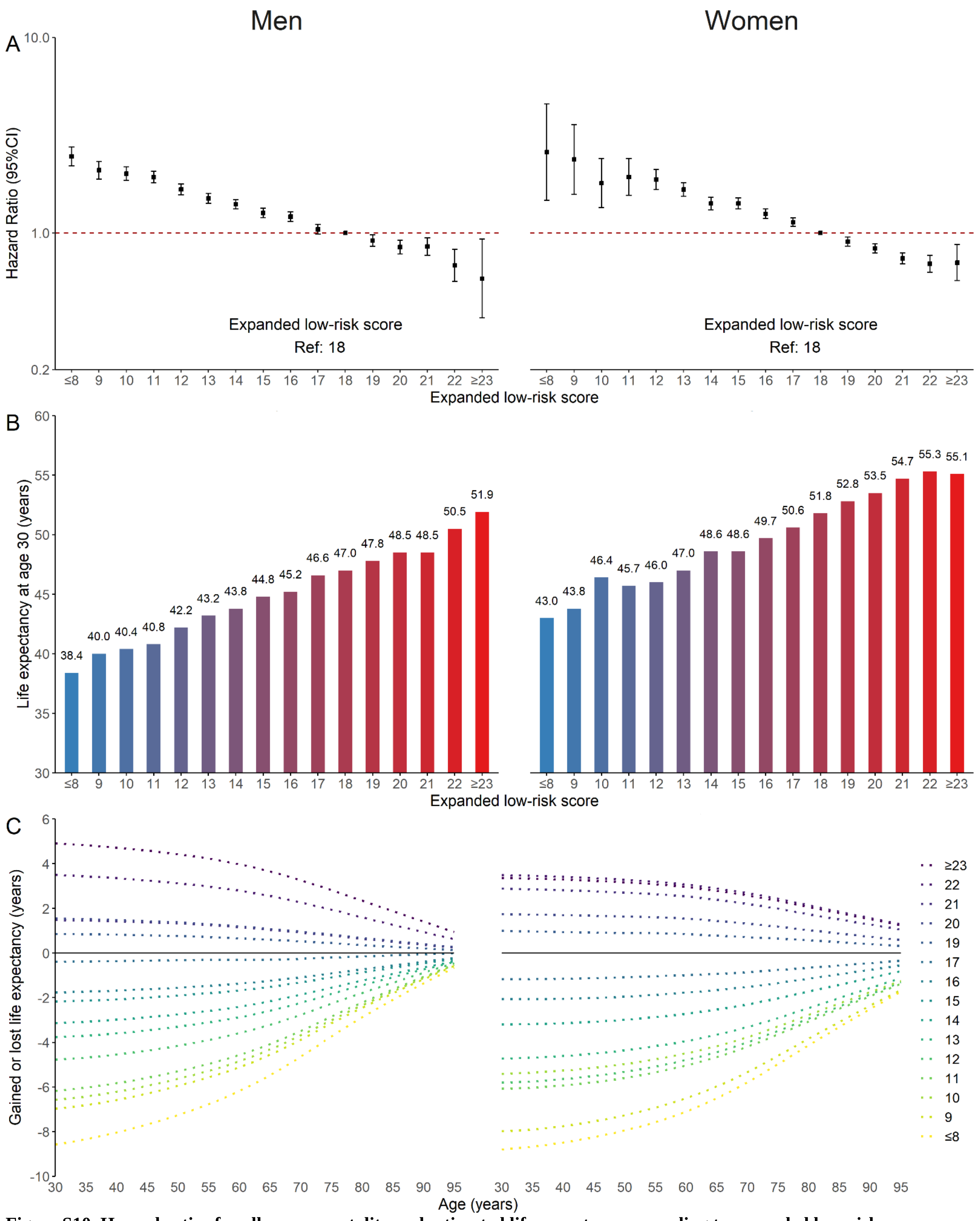

Figure S10. Hazard ratios for all-cause mortality and estimated life expectancy according to expanded low-risk score. (A) Hazard ratios (95%CIs) for all-cause mortality; (B) Estimated life expectancy at age 30; (C) Estimated gained or lost age-specific life expectancy from adopting low-risk lifestyle as compared to the median score group.

To calculate the expanded score for low-risk lifestyle factors, we graded the categories of each lifestyle factor from 1 (least healthy) to 5 (most healthy) based on the associations between lifestyle factors and all-cause mortality (Table 1), and summed the points across all five lifestyle factors. The scores assigned to each category are listed in parentheses below:

- Smoking: never (5), former (4), current 1-9 cigarettes/day (3), current 10-19 cigarettes/day (2), current ≥20 cigarettes/day (1)
- Alcohol intake: Less than daily (4), current 1-14 g/d (5), current 15-29 g/d (3), current 30-59 g/d (2), current ≥60 g/d or former drinkers (1)
- Dietary score: 0-1 (1), 2 (2), 3 (3), 4 (4), 5 (5)
- Physical activity: Quintile 1 (1), Quintile 2 (2), Quintile 3 (3), Quintile 4 (4), Quintile 5 (5)
- Body shape: BMI <18.5 kg/m^2 (2), BMI 18.5-27.9 kg/m^2, waist circumference <90 cm (men)/85 cm (women) (5), BMI 18.5-27.9 kg/m^2, waist circumference ≥90 cm (men)/85 cm (women) (4), BMI ≥28.0 kg/m^2, waist circumference <90 cm (men)/85 cm (women) (3), BMI ≥28.0 kg/m^2, waist circumference ≥90 cm (men)/85 cm (women) (1)
